# Risk factors for COVID-19 death in a population cohort study from the Western Cape Province, South Africa

**DOI:** 10.1093/cid/ciaa1198

**Published:** 2020-08-29

**Authors:** Andrew Boulle, Mary-Ann Davies, Hannah Hussey, Muzzammil Ismail, Erna Morden, Ziyanda Vundle, Virginia Zweigenthal, Hassan Mahomed, Masudah Paleker, David Pienaar, Yamanya Tembo, Charlene Lawrence, Washiefa Isaacs, Hlengani Mathema, Derick Allen, Taryn Allie, Jamy-Lee Bam, Kasturi Buddiga, Pierre Dane, Alexa Heekes, Boitumelo Matlapeng, Themba Mutemaringa, Luckmore Muzarabani, Florence Phelanyane, Rory Pienaar, Catherine Rode, Mariette Smith, Nicki Tiffin, Nesbert Zinyakatira, Carol Cragg, Frederick Marais, Vanessa Mudaly, Jacqueline Voget, Jody Davids, Francois Roodt, Nellis van Zyl Smit, Alda Vermeulen, Kevin Adams, Gordon Audley, Kathleen Bateman, Peter Beckwith, Marc Bernon, Dirk Blom, Linda Boloko, Jean Botha, Adam Boutall, Sean Burmeister, Lydia Cairncross, Gregory Calligaro, Cecilia Coccia, Chadwin Corin, Remy Daroowala, Joel A Dave, Elsa De Bruyn, Martin De Villiers, Mimi Deetlefs, Sipho Dlamini, Thomas Du Toit, Wilhelm Endres, Tarin Europa, Graham Fieggan, Anthony Figaji, Petro Frankenfeld, Elizabeth Gatley, Phindile Gina, Evashan Govender, Rochelle Grobler, Manqoba Vusumuzi Gule, Christoff Hanekom, Michael Held, Alana Heynes, Sabelo Hlatswayo, Bridget Hodkinson, Jeanette Holtzhausen, Shakeel Hoosain, Ashely Jacobs, Miriam Kahn, Thania Kahn, Arvin Khamajeet, Joubin Khan, Riaasat Khan, Alicia Khwitshana, Lauren Knight, Sharita Kooverjee, Rene Krogscheepers, Jean Jacque Kruger, Suzanne Kuhn, Kim Laubscher, John Lazarus, Jacque Le Roux, Scott Lee Jones, Dion Levin, Gary Maartens, Thina Majola, Rodgers Manganyi, David Marais, Suzaan Marais, Francois Maritz, Deborah Maughan, Simthandile Mazondwa, Luyanda Mbanga, Nomonde Mbatani, Bulewa Mbena, Graeme Meintjes, Marc Mendelson, Ernst Möller, Allison Moore, Babalwa Ndebele, Marc Nortje, Ntobeko Ntusi, Funeka Nyengane, Chima Ofoegbu, Nectarios Papavarnavas, Jonny Peter, Henri Pickard, Kent Pluke, Peter J Raubenheimer, Gordon Robertson, Julius Rozmiarek, A Sayed, Matthias Scriba, Hennie Sekhukhune, Prasun Singh, Elsabe Smith, Vuyolwethu Soldati, Cari Stek, Robert van den berg, Le Roux van der Merwe, Pieter Venter, Barbra Vermooten, Gerrit Viljoen, Santhuri Viranna, Jonno Vogel, Nokubonga Vundla, Sean Wasserman, Eddy Zitha, Vanessa Lomas-Marais, Annie Lombard, Katrin Stuve, Werner Viljoen, De Vries Basson, Sue Le Roux, Ethel Linden-Mars, Lizanne Victor, Mark Wates, Elbe Zwanepoel, Nabilah Ebrahim, Sa'ad Lahri, Ayanda Mnguni, Thomas Crede, Martin de Man, Katya Evans, Clint Hendrikse, Jonathan Naude, Moosa Parak, Patrick Szymanski, Candice Van Koningsbruggen, Riezaah Abrahams, Brian Allwood, Christoffel Botha, Matthys Henndrik Botha, Alistair Broadhurst, Dirkie Claasen, Che Daniel, Riyaadh Dawood, Marie du Preez, Nicolene Du Toit, Kobie Erasmus, Coenraad F N Koegelenberg, Shiraaz Gabriel, Susan Hugo, Thabiet Jardine, Clint Johannes, Sumanth Karamchand, Usha Lalla, Eduard Langenegger, Eize Louw, Boitumelo Mashigo, Nonte Mhlana, Chizama Mnqwazi, Ashley Moodley, Desiree Moodley, Saadiq Moolla, Abdurasiet Mowlana, Andre Nortje, Elzanne Olivier, Arifa Parker, Chané Paulsen, Hans Prozesky, Jacques Rood, Tholakele Sabela, Neshaad Schrueder, Nokwanda Sithole, Sthembiso Sithole, Jantjie J Taljaard, Gideon Titus, Tian Van Der Merwe, Marije van Schalkwyk, Luthando Vazi, Abraham J Viljoen, Mogamat Yazied Chothia, Vanessa Naidoo, Lee Alan Wallis, Mumtaz Abbass, Juanita Arendse, Rizqa Armien, Rochelle Bailey, Muideen Bello, Rachel Carelse, Sheron Forgus, Nosi Kalawe, Saadiq Kariem, Mariska Kotze, Jonathan Lucas, Juanita McClaughlin, Kathleen Murie, Leilah Najjaar, Liesel Petersen, James Porter, Melanie Shaw, Dusica Stapar, Michelle Williams, Linda Aldum, Natacha Berkowitz, Raakhee Girran, Kevin Lee, Lenny Naidoo, Caroline Neumuller, Kim Anderson, Kerrin Begg, Lisa Boerlage, Morna Cornell, Renée de Waal, Lilian Dudley, René English, Jonathan Euvrard, Pam Groenewald, Nisha Jacob, Heather Jaspan, Emma Kalk, Naomi Levitt, Thoko Malaba, Patience Nyakato, Gabriela Patten, Helen Schneider, Maylene Shung King, Priscilla Tsondai, James Van Duuren, Nienke van Schaik, Lucille Blumberg, Cheryl Cohen, Nelesh Govender, Waasila Jassat, Tendesayi Kufa, Kerrigan McCarthy, Lynn Morris, Nei-yuan Hsiao, Ruan Marais, Jon Ambler, Olina Ngwenya, Richard Osei-Yeboah, Leigh Johnson, Reshma Kassanjee, Tsaone Tamuhla

**Affiliations:** 1 Health Impact Assessment, Western Cape Government: Health; 2 Centre for Infectious Disease Epidemiology and Research, School of Public Health and Family Medicine, University of Cape Town; 3 School of Public Health and Family Medicine, University of Cape Town; 4 Division of Health Systems and Public Health, Department of Global Health, Faculty of Medicine and Health Sciences, Stellenbosch University; 5 Metro Health Services, Western Cape Government: Health; 6 Rural Health Services, Western Cape Government: Health; 7 Communicable Disease Sub-Directorate, Western Cape Government: Health; 8 National Institute for Communicable Diseases, National Health Laboratory Service, South Africa; 9 Wellcome Centre for Infectious Disease Research in Africa, University of Cape Town; 10 Division of Computational Biology, University of Cape Town; 11 Health Programmes Directorate, Western Cape Government: Health; 12 Faculty of Health Sciences, North West University; 13 George Hospital, Western Cape Government: Health; 14 Groote Schuur Hospital, Western Cape Government: Health; 15 Department of Medicine, University of Cape Town; 16 Department of Surgery, University of Cape Town; 17 Department of Radiology, University of Cape Town; 18 Karl Bremer Hospital, Western Cape Government: Health; 19 Khayelitsha District Hospital, Western Cape Government: Health; 20 Mitchells Plain and Heideveld Hospitals, Western Cape Government: Health; 21 Tygerberg Hospital, Western Cape Government: Health; 22 Department of Medicine, Stellenbosch University; 23 Department of Obstetrics and Gyneacology, Stellenbosch University; 24 Emergency Medical Services, Western Cape Government; 25 Western Cape Government: Health; 26 City Health, Community Services and Health, City of Cape Town; 27 South African Medical Research Council Burden of Disease Research Unit; 28 School of Public Health, University of the Western Cape; 29 School of Pathology, University of the Witwatersrand and School of Pathology, University of Cape Town; 30 National Health Laboratory Service and Division of Virology, School of Pathology, University of Cape Town; 31 Division of Emergency Medicine, University of Cape Town; 32 Division of Immunology and Institute of Infectious Diseases and Molecular Medicine, University of Cape Town; 33 University of Pretoria; 34 School of Public Health, University of Witwatersrand; 35 University of Witwatersrand, South African Medical Research Council Antibody Immunity Research Unit and the Centre for the AIDS Programme in South Africa (CAPRISA)

**Keywords:** COVID-19, HIV, tuberculosis, sub-Saharan Africa, antiretroviral

## Abstract

**Background:**

Risk factors for COVID-19 death in sub-Saharan Africa and the effects of HIV and tuberculosis on COVID-19 outcomes are unknown.

**Methods:**

We conducted a population cohort study using linked data from adults attending public sector health facilities in the Western Cape, South Africa. We used Cox-proportional hazards models adjusted for age, sex, location and comorbidities to examine the association between HIV, tuberculosis and COVID-19 death from 1 March-9 June 2020 among (i) public sector “active patients” (≥1 visit in the 3 years before March 2020), (ii) laboratory-diagnosed COVID-19 cases and (iii) hospitalized COVID-19 cases. We calculated the standardized mortality ratio (SMR) for COVID-19 comparing HIV positive vs. negative adults using modelled population estimates.

**Results:**

Among 3,460,932 patients (16% HIV positive), 22,308 were diagnosed with COVID-19, of whom 625 died. COVID-19 death was associated with male sex, increasing age, diabetes, hypertension and chronic kidney disease. HIV was associated with COVID-19 mortality (adjusted hazard ratio [aHR] 2.14; 95% confidence interval [CI] 1.70-2.70), with similar risks across strata of viral load and immunosuppression. Current and previous tuberculosis were associated with COVID-19 death (aHR [95%CI] 2.70 [1.81-4.04] and 1.51 [1.18-1.93] respectively). The SMR for COVID-19 death associated with HIV was 2.39 (95%CI 1.96-2.86); population attributable fraction 8.5% (95%CI 6.1-11.1).

**Conclusion:**

While our findings may over-estimate HIV- and tuberculosis-associated COVID-19 mortality risks due to residual confounding, both HIV and current tuberculosis were independently associated with increased COVID-19 mortality. The associations between age, sex and other comorbidities and COVID-19 mortality were similar to other settings.

