# Risk Factors for Coronavirus Disease 2019 (COVID-19) Death in a Population Cohort Study from the Western Cape Province, South Africa

**DOI:** 10.1093/cid/ciaa1198

**Published:** 2020-08-29

**Authors:** Western Cape Department of Health in collaboration with the National Institute for Communicable Diseases, South Africa, Andrew Boulle, Mary-Ann Davies, Hannah Hussey, Muzzammil Ismail, Erna Morden, Ziyanda Vundle, Virginia Zweigenthal, Hassan Mahomed, Masudah Paleker, David Pienaar, Yamanya Tembo, Charlene Lawrence, Washiefa Isaacs, Hlengani Mathema, Derick Allen, Taryn Allie, Jamy-Lee Bam, Kasturi Buddiga, Pierre Dane, Alexa Heekes, Boitumelo Matlapeng, Themba Mutemaringa, Luckmore Muzarabani, Florence Phelanyane, Rory Pienaar, Catherine Rode, Mariette Smith, Nicki Tiffin, Nesbert Zinyakatira, Carol Cragg, Frederick Marais, Vanessa Mudaly, Jacqueline Voget, Jody Davids, Francois Roodt, Nellis van Zyl Smit, Alda Vermeulen, Kevin Adams, Gordon Audley, Kathleen Bateman, Peter Beckwith, Marc Bernon, Dirk Blom, Linda Boloko, Jean Botha, Adam Boutall, Sean Burmeister, Lydia Cairncross, Gregory Calligaro, Cecilia Coccia, Chadwin Corin, Remy Daroowala, Joel A Dave, Elsa De Bruyn, Martin De Villiers, Mimi Deetlefs, Sipho Dlamini, Thomas Du Toit, Wilhelm Endres, Tarin Europa, Graham Fieggan, Anthony Figaji, Petro Frankenfeld, Elizabeth Gatley, Phindile Gina, Evashan Govender, Rochelle Grobler, Manqoba Vusumuzi Gule, Christoff Hanekom, Michael Held, Alana Heynes, Sabelo Hlatswayo, Bridget Hodkinson, Jeanette Holtzhausen, Shakeel Hoosain, Ashely Jacobs, Miriam Kahn, Thania Kahn, Arvin Khamajeet, Joubin Khan, Riaasat Khan, Alicia Khwitshana, Lauren Knight, Sharita Kooverjee, Rene Krogscheepers, Jean Jacque Kruger, Suzanne Kuhn, Kim Laubscher, John Lazarus, Jacque Le Roux, Scott Lee Jones, Dion Levin, Gary Maartens, Thina Majola, Rodgers Manganyi, David Marais, Suzaan Marais, Francois Maritz, Deborah Maughan, Simthandile Mazondwa, Luyanda Mbanga, Nomonde Mbatani, Bulewa Mbena, Graeme Meintjes, Marc Mendelson, Ernst Möller, Allison Moore, Babalwa Ndebele, Marc Nortje, Ntobeko Ntusi, Funeka Nyengane, Chima Ofoegbu, Nectarios Papavarnavas, Jonny Peter, Henri Pickard, Kent Pluke, Peter J Raubenheimer, Gordon Robertson, Julius Rozmiarek, A Sayed, Matthias Scriba, Hennie Sekhukhune, Prasun Singh, Elsabe Smith, Vuyolwethu Soldati, Cari Stek, Robert van den berg, Le Roux van der Merwe, Pieter Venter, Barbra Vermooten, Gerrit Viljoen, Santhuri Viranna, Jonno Vogel, Nokubonga Vundla, Sean Wasserman, Eddy Zitha, Vanessa Lomas-Marais, Annie Lombard, Katrin Stuve, Werner Viljoen, De Vries Basson, Sue Le Roux, Ethel Linden-Mars, Lizanne Victor, Mark Wates, Elbe Zwanepoel, Nabilah Ebrahim, Sa’ad Lahri, Ayanda Mnguni, Thomas Crede, Martin de Man, Katya Evans, Clint Hendrikse, Jonathan Naude, Moosa Parak, Patrick Szymanski, Candice Van Koningsbruggen, Riezaah Abrahams, Brian Allwood, Christoffel Botha, Matthys Henndrik Botha, Alistair Broadhurst, Dirkie Claasen, Che Daniel, Riyaadh Dawood, Marie du Preez, Nicolene Du Toit, Kobie Erasmus, Coenraad F N Koegelenberg, Shiraaz Gabriel, Susan Hugo, Thabiet Jardine, Clint Johannes, Sumanth Karamchand, Usha Lalla, Eduard Langenegger, Eize Louw, Boitumelo Mashigo, Nonte Mhlana, Chizama Mnqwazi, Ashley Moodley, Desiree Moodley, Saadiq Moolla, Abdurasiet Mowlana, Andre Nortje, Elzanne Olivier, Arifa Parker, Chané Paulsen, Hans Prozesky, Jacques Rood, Tholakele Sabela, Neshaad Schrueder, Nokwanda Sithole, Sthembiso Sithole, Jantjie J Taljaard, Gideon Titus, Tian Van Der Merwe, Marije van Schalkwyk, Luthando Vazi, Abraham J Viljoen, Mogamat Yazied Chothia, Vanessa Naidoo, Lee Alan Wallis, Mumtaz Abbass, Juanita Arendse, Rizqa Armien, Rochelle Bailey, Muideen Bello, Rachel Carelse, Sheron Forgus, Nosi Kalawe, Saadiq Kariem, Mariska Kotze, Jonathan Lucas, Juanita McClaughlin, Kathleen Murie, Leilah Najjaar, Liesel Petersen, James Porter, Melanie Shaw, Dusica Stapar, Michelle Williams, Linda Aldum, Natacha Berkowitz, Raakhee Girran, Kevin Lee, Lenny Naidoo, Caroline Neumuller, Kim Anderson, Kerrin Begg, Lisa Boerlage, Morna Cornell, Renée de Waal, Lilian Dudley, René English, Jonathan Euvrard, Pam Groenewald, Nisha Jacob, Heather Jaspan, Emma Kalk, Naomi Levitt, Thoko Malaba, Patience Nyakato, Gabriela Patten, Helen Schneider, Maylene Shung King, Priscilla Tsondai, James Van Duuren, Nienke van Schaik, Lucille Blumberg, Cheryl Cohen, Nelesh Govender, Waasila Jassat, Tendesayi Kufa, Kerrigan McCarthy, Lynn Morris, Nei-yuan Hsiao, Ruan Marais, Jon Ambler, Olina Ngwenya, Richard Osei-Yeboah, Leigh Johnson, Reshma Kassanjee, Tsaone Tamuhla

**Affiliations:** 1 Health Impact Assessment, Western Cape Government: Health; 2 Centre for Infectious Disease Epidemiology and Research, School of Public Health and Family Medicine, University of Cape Town; 3 School of Public Health and Family Medicine, University of Cape Town; 4 Division of Health Systems and Public Health, Department of Global Health, Faculty of Medicine and Health Sciences, Stellenbosch University; 5 Metro Health Services, Western Cape Government: Health; 6 Rural Health Services, Western Cape Government: Health; 7 Communicable Disease Sub-Directorate, Western Cape Government: Health; 8 National Institute for Communicable Diseases, National Health Laboratory Service, South Africa; 9 Wellcome Centre for Infectious Disease Research in Africa, University of Cape Town; 10 Division of Computational Biology, University of Cape Town; 11 Health Programmes Directorate, Western Cape Government: Health; 12 Faculty of Health Sciences, North West University; 13 George Hospital, Western Cape Government: Health; 14 Groote Schuur Hospital, Western Cape Government: Health; 15 Department of Surgery, University of Cape Town; 16 Department of Medicine, University of Cape Town; 17 Department of Radiology, University of Cape Town; 18 Karl Bremer Hospital, Western Cape Government: Health; 19 Khayelitsha District Hospital, Western Cape Government: Health; 20 Mitchells Plain and Heideveld Hospitals, Western Cape Government: Health; 21 Tygerberg Hospital, Western Cape Government: Health; 22 Department of Medicine, Stellenbosch University; 23 Department of Obstetrics and Gyneacology, Stellenbosch University; 24 Emergency Medical Services, Western Cape Government; 25 Western Cape Government: Health; 26 City Health, Community Services and Health, City of Cape Town; 27 South African Medical Research Council Burden of Disease Research Unit; 28 School of Public Health, University of the Western Cape; 29 School of Pathology, University of the Witwatersrand and School of Pathology, University of Cape Town; 30 National Health Laboratory Service and Division of Virology, School of Pathology, University of Cape Town; 31 Division of Emergency Medicine, University of Cape Town; 32 Division of Immunology and Institute of Infectious Diseases and Molecular Medicine, University of Cape Town; 33 University of Pretoria; 34 School of Public Health, University of Witwatersrand; 35 University of Witwatersrand, South African Medical Research Council Antibody Immunity Research Unit and the Centre for the AIDS Programme in South Africa (CAPRISA)

**Keywords:** COVID-19, HIV, tuberculosis, sub-Saharan Africa, antiretroviral

## Abstract

**Background:**

Risk factors for coronavirus disease 2019 (COVID-19) death in sub-Saharan Africa and the effects of human immunodeficiency virus (HIV) and tuberculosis on COVID-19 outcomes are unknown.

**Methods:**

We conducted a population cohort study using linked data from adults attending public-sector health facilities in the Western Cape, South Africa. We used Cox proportional hazards models, adjusted for age, sex, location, and comorbidities, to examine the associations between HIV, tuberculosis, and COVID-19 death from 1 March to 9 June 2020 among (1) public-sector “active patients” (≥1 visit in the 3 years before March 2020); (2) laboratory-diagnosed COVID-19 cases; and (3) hospitalized COVID-19 cases. We calculated the standardized mortality ratio (SMR) for COVID-19, comparing adults living with and without HIV using modeled population estimates.

**Results:**

Among 3 460 932 patients (16% living with HIV), 22 308 were diagnosed with COVID-19, of whom 625 died. COVID-19 death was associated with male sex, increasing age, diabetes, hypertension, and chronic kidney disease. HIV was associated with COVID-19 mortality (adjusted hazard ratio [aHR], 2.14; 95% confidence interval [CI], 1.70–2.70), with similar risks across strata of viral loads and immunosuppression. Current and previous diagnoses of tuberculosis were associated with COVID-19 death (aHR, 2.70 [95% CI, 1.81–4.04] and 1.51 [95% CI, 1.18–1.93], respectively). The SMR for COVID-19 death associated with HIV was 2.39 (95% CI, 1.96–2.86); population attributable fraction 8.5% (95% CI, 6.1–11.1).

**Conclusions:**

While our findings may overestimate HIV- and tuberculosis-associated COVID-19 mortality risks due to residual confounding, both living with HIV and having current tuberculosis were independently associated with increased COVID-19 mortality. The associations between age, sex, and other comorbidities and COVID-19 mortality were similar to those in other settings.

## Teaser

In this population cohort of 3.5 million public-sector patients in South Africa, increased coronavirus disease 2019 (COVID-19) mortality was associated with human immunodeficiency virus (HIV), previous and current tuberculosis, older age, male sex, diabetes, hypertension, and chronic kidney disease.

The effects of the intersecting pandemics of human immunodeficiency virus (HIV), tuberculosis, and coronavirus disease 2019 (COVID-19) in sub-Saharan Africa are unknown. Studies to date suggest no increased risk of adverse outcomes for COVID-19 in patients living with HIV and coinfected with tuberculosis, but these are small studies from Europe and North America, often limited to hospitalized patients, and may not be relevant to sub-Saharan Africa, where people living with HIV (PLWH) are younger, with different comorbidities, frequently including tuberculosis [1–8]. PLWH may experience more severe COVID-19 disease due to HIV-related immune suppression, which may be exacerbated by transient immune deficiency from coronaviruses [9, 10]. In support of this hypothesis, a large UK cohort study reported an increased risk of COVID-19 death in those with immunosuppressive comorbidities, including PLWH [8]. However, 2 factors may reduce the risk of severe COVID-19 in PLWH: dysfunctional immunity may lessen a virus-induced cytokine storm [11, 12], and some antiretroviral drugs (tenofovir and some protease inhibitors) have in vitro activity against coronaviruses, with better outcomes reported for PLWH receiving tenofovir disoproxil fumarate (TDF) versus other antiretrovirals [12, 13]. Tuberculosis may exacerbate COVID-19, with impaired immune responses and increased angiotensin converting enzyme 2 receptor expression in respiratory epithelial cells, while COVID-19 pneumonia may enhance tuberculosis progression [14–17].

It is important to establish whether HIV and tuberculosis increase risks of COVID-19 death, so that patients with these conditions can be provided with augmented prevention and potential therapeutic interventions. We used linked data from adults attending public-sector health facilities in the Western Cape Province, South Africa, to identify factors associated with COVID-19 death.

## METHODS

### Study Design

We conducted a cohort study using deidentified data from the Western Cape Provincial Health Data Centre (WCPHDC) of public-sector patients aged ≥20 years with documented sex and not known to have died before 1 March 2020 (before the first diagnosed COVID-19 case in South Africa, and several weeks before the first documented COVID-19 death) and included all follow-up through 9 June 2020. The outcome was COVID-19–associated death. Our main analysis examined the risk of COVID-19 death in the general population, so all patients were included irrespective of severe acute respiratory syndrome coronavirus 2 (SARS-CoV-2) testing. The study was approved by the University of Cape Town and Stellenbosch University Health Research Ethics Committees and the Western Cape Province Department of Health. Individual informed consent requirement was waived for this secondary analysis of deidentified data.

### Study Population and Data Sources

The Western Cape has nearly 7 million inhabitants, of whom ~520 000 are PLWH, with >90% of them dependent on public-sector health services. The WCPHDC has been described in detail [18]. Briefly, WCPHDC consolidates administrative, laboratory, and pharmacy data from routine electronic clinical information systems used in all public-sector health facilities, with linkage through a unique identifier. Multiple data sources are triangulated to enumerate health conditions such as diabetes mellitus, hypertension, tuberculosis, and HIV, with a label of high- or moderate-certainty evidence assigned for each inferred condition (Supplementary Table 1). High-certainty evidence of HIV comprises a positive HIV diagnostic test, HIV RNA test, triple antiretroviral therapy (ART), and/or registration in the HIV disease management system; moderate certainty is assigned for those with only a CD4 count measure, 2 antiretroviral drugs prescribed (previously used for vertical HIV transmission prevention), and/or an International Classification of Disease, Tenth Revision, diagnosis code of HIV. HIV testing coverage is high, as >90% of PLWH know their HIV diagnosis [19]. High-certainty evidence of tuberculosis comprised laboratory evidence of *Mycobacterium tuberculosis* infection (any anatomical site, using Xpert RIF/MTB, microscopy, and culture), registration on the electronic tuberculosis registers, combination tuberculosis treatment, and/or admission to a tuberculosis hospital. Comorbidities were based on high- or moderate-certainty evidence but were restricted to high-certainty evidence in sensitivity analyses. The virologic, immunologic, and ART statuses of PLWH on 1 March 2020 were categorized, based on the most recent measures, as “confirmed virologically suppressed on ART” (HIV RNA < 1000 copies/ml in the last 15 months and ART dispensed in the last 6 months), “likely virologically suppressed on ART” (HIV RNA < 1000 copies/ml 15–24 months previously or HIV RNA < 1000 copies/ml > 24 months previously if ART dispensed in the last 6 months), “viremic or immunosuppressed” (HIV RNA > 1000 copies/ml in the last 15 months or CD4 count < 200 cells/µl within 18 months before March 2020), or “unknown.” Until January 2020, adult first-line ART was TDF, emtricitabine/lamivudine, and efavirenz, with abacavir replacing TDF for patients with kidney disease; zidovudine plus an emtricitabine/protease inhibitor were used for second-line ART for most patients. Dolutegravir was introduced in first- and second-line ART in January 2020. Diabetic control was categorized according to glycosylated hemoglobin (HbA1c) measurements within the last 2 years, with <7% indicating diabetes was controlled, 7–8.9% indicating diabetes was poorly controlled, and ≥9% indicating diabetes was uncontrolled.

### COVID-19 Diagnosis

All COVID-19 diagnoses were based on a positive SARS-CoV-2 polymerase chain reaction test. Testing was available for all patients with COVID-19 symptoms until 1 June 2020; thereafter, public-sector laboratory testing was restricted to patients requiring admission, aged >55 years, or with comorbidities, due to the temporary limited testing capacity. Hospital admissions and all deaths in SARS-CoV-2–positive cases are recorded and reviewed daily.

### Statistical Analysis

We used Cox proportional hazards models, adjusted for age, sex, and other comorbidities, to examine the associations between HIV, tuberculosis, and COVID-19 death among (1) all public-sector patients with ≥1 health visit in the 3 years before 1 March 2020 (considered “active patients”); (2) laboratory-diagnosed COVID-19 cases; and (3) hospitalized COVID-19 cases. We adjusted for location within Cape Town versus the rest of the province, and by the subdistrict of residence within Cape Town, to account for geographical variation in infection rates and as a proxy for socioeconomic status. Patients were censored on their date of death if deceased without a COVID-19 diagnosis, or on 9 June 2020, whichever was earliest. The database closure was 7 days later to allow for death reporting delays. For the analysis of COVID-19 deaths in laboratory-diagnosed cases, we included cases diagnosed before 1 June 2020, when testing was available for all patients with COVID-19 symptoms, but included all patients diagnosed by 9 June 2020 in the sensitivity analysis. The proportional-hazard assumption was assessed with Schoenfeld residuals [20]. All analyses were conducted using Stata 15.1.

We also calculated the standardized mortality ratio (SMR) of the actual number of COVID-19 deaths in PLWH versus the number that would be expected if PLWH had the same risk of COVID-19 death as people living without HIV of the same age and sex. We used data on the age, sex, and HIV status of all COVID-19 deaths (public and private sector) and the Thembisa Western Cape HIV model to estimate the Western Cape population size and HIV prevalence, by age and sex, in 2020 [21]. We calculated 95% confidence intervals (CI) for the SMR using 1000 bootstrap replications (see Supplementary Appendix 2).

Since individual socioeconomic status and some comorbidities are not recorded in WCPHDC, we calculated E-values to determine the minimum strength of association that an unmeasured confounder (eg, raised body mass index [BMI] or socioeconomic status) would need to have with HIV/ tuberculosis and COVID-19 death to fully account for any association between HIV/tuberculosis and COVID-19 death [22]. We conducted a quantitative bias analysis to assess the impact of potential confounding by obesity on an association between HIV and COVID-19 death.

## RESULTS

### Patient Characteristics

Among 3 460 932 active patients aged ≥20 years on 1 March 2020, 22 308 were diagnosed with COVID-19, of whom 625 (2.8%) died (Table 1). Among COVID-19 cases, 69% were diagnosed and 67% of deaths occurred before the change in testing criteria (1 June). The proportion of men was lower among COVID-19 cases versus noncases (31% vs 42%, respectively), likely due to initial cases being among essential workers in retail and manufacturing sectors that predominantly employ women. The proportion of women peaked (76%) in Week 5 of the epidemic, declining thereafter. Diabetes and hypertension were common in all patients, with higher prevalences among COVID-19 cases than noncases (diabetes, 14% vs 8%, respectively; hypertension, 23% vs 16%, respectively), while proportions of other comorbidities (as listed below) were similar. The COVID-19 patients who died were older than those who survived (median age, 63 years [interquartile range, 54–71] vs 37 [interquartile range, 30–48], respectively); several comorbidities were more common among the deceased patients than survivors (diabetes, 60% vs 14%, respectively; hypertension, 58% vs 23%, respectively; chronic kidney disease, 18% vs 2%, respectively; chronic pulmonary disease/asthma, 13% vs 7%, respectively; current tuberculosis, 4% vs 1%, respectively; previous tuberculosis, 14% vs 8%, respectively).

**Table 1.** Patient Characteristics

	All public-sector patients			All public-sector SARS-CoV-2 cases diagnosed before 1 June 2020^a^		Hospitalized public-sector SARS-CoV-2 cases	
	No diagnosed COVID-19, n = 3 438 624	COVID-19 cases, not deceased, n = 21 683	COVID-19 cases, deceased, n = 625	COVID-19 cases, not deceased, n = 14 693	COVID-19 cases, deceased, n = 510	COVID-19 cases, not deceased, n = 2428	COVID-19 cases, deceased, n = 550
Sex							
Female	1 983 480 (58%)	14 916 (69%)	340 (54%)	10 388 (71%)	281 (55%)	1546 (64%)	302 (55%)
Male	1 455 144 (42%)	6767 (31%)	285 (46%)	4305 (29%)	229 (45%)	882 (36%)	248 (49%)
Age							
20–39 years	1 913 786 (56%)	1164 (54%)	46 (7%)	8653 (59%)	35 (7%)	804 (33%)	45 (8%)
40–49 years	604 976 (18%)	4515 (21%)	63 (10%)	2991 (20%)	51 (10%)	472 (19%)	56 (10%)
50–59 years	447 739 (13%)	3227 (15%)	162 (26%)	1881 (13%)	137 (27%)	523 (2 x 1%)	142 (26%)
60–69 years	276 082 (8%)	1423 (7%)	178 (28%)	739 (5%)	150 (29%)	360 (15%)	158 (29%)
≥70 years	196 035 (6%)	878 (4%)	176 (28%)	429 (3%)	137 (27%)	269 (11%)	149 (27%)
Diabetes							
None	3 177 088 (92%)	18 581 (86%)	253 (40%)	12 921 (88%)	212 (42%)	1549 (64%)	225 (41%)
Diabetes HbA1c <7%	45 054 (1%)	491 (2%)	58 (9%)	278 (2%)	43 (8%)	149 (6%)	54 (10%)
Diabetes HbA1c 7–8.9%	47 211 (1%)	582 (3%)	94 (15%)	309 (2%)	79 (15%)	164 (7%)	85 (15%)
Diabetes HbA1c ≥9%	65 639 (2%)	1086 (5%)	158 (25%)	640 (4%)	125 (25%)	375 (15%)	136 (25%)
Diabetes, no HbA1c measurement	103 632 (3%)	943 (4%)	62 (10%)	545 (4%)	51 (10%)	191 (8%)	50 (9%)
Other noncommunicable diseases							
Hypertension	563 908 (16%)	4910 (23%)	362 (58%)	2972 (20%)	293 (57%)	948 (39%)	319 (58%)
Chronic kidney disease	61 667 (2%)	494 (2%)	111 (18%)	250 (2%)	92 (18%)	168 (7%)	101 (18%)
Chronic pulmonary disease/asthma	192 587 (6%)	1577 (7%)	84 (13%)	949 (6%)	77 (15%)	355 (15%)	78 (14%)
Tuberculosis							
Never tuberculosis	3 097 483 (90%)	19 668 (91%)	512 (82%)	13 330 (91%)	414 (81%)	2061 (85%)	448 (81%)
Previous tuberculosis	286 889 (8%)	1698 (8%)	87 (14%)	1180 (8%)	74 (15%)	244 (10%)	77 (14%)
Current tuberculosis	54 252 (2%)	317 (1%)	26 (4%)	213 (1%)	22(4%)	123 (5%)	25 (5%)
HIV							
Negative	2 902 050 (84%)	17 820 (82%)	510 (82%)	11 893 (81%)	415 (81%)	1932 (80%)	445 (81%)
Positive	536 574 (16%)	3863 (18%)	115 (18%)	2800 (19%)	95 (19%)	496 (20%)	105 (19%)
VL <1000 copies/ml (last 15 mo) and ART script (last 6 mo)	240 048 (45%)	2332 (60%)	71 (62%)	1726 (62%)	57 (60%)	289 (58%)	68 (65%)
VL <1000 copies/ml (2yr to 15 mo prior), or ART script (last 6 mo) and VL <1000 copies/ml > 2yr prior	68 871 (13%)	409 (11%)	11 (10%)	283 (10%)	9 (9%)	46 (9%)	11 (10%)
VL ≥1000 copies/ml (last 15 mo) or CD4 count < 200 cells/µl (last 18 mo)	40 974 (8%)	209 (5%)	12 (10%)	150 (5%)	10 (11%)	58 (12%)	9 (9%)
No VL (last 15 mo); CD4 count ≥ 200 cells/µl or unknown (last 18 mo)	186 681 (35%)	913 (24%)	21 (18%)	641 (23%)	19 (20%)	103 (21%)	17 (16%

Data are of (1) Western Cape active patients aged ≥20 years in the public sector (public-sector health-care visit in last 3 years before 1 March 2020), according to COVID-19 outcome; (2) COVID-19 cases in active patients; and (3) hospitalized COVID-19 cases in active patients. Column percentages may add up to >100% due to rounding.

Abbreviations: ART, antiretroviral therapy; COVID-19, coronavirus disease 2019; HbA1c, glycosylated haemoglobin; HIV, human immunodeficiency virus; SARS-CoV-2, severe acute respiratory syndrome coronavirus 2; VL, viral load.

^a^Analysis limited to cases diagnosed before 1 June 2020, when testing criteria changed with public-sector tests being limited to patients >55 year of age or with comorbidities.

### Patients With and Without HIV

Although the proportion of PLWH was similar among surviving and deceased COVID-19 cases, a greater proportion of COVID-19 deaths were in patients aged <50 years in those living with versus without HIV (39% vs 13%, respectively; Table 2). A substantial proportion of PLWH who died from COVID-19 had diabetes (50%) and hypertension (42%); however, these conditions were more common in deceased people living without HIV (62% for each condition). Current and previous tuberculosis were more frequent in PLWH, irrespective of COVID-19 survival status, with 14% and 37% of COVID-19 deceased cases in PLWH having current and/or previous tuberculosis, respectively.

**Table 2.** Patient Characteristics by Human Immunodeficiency Virus Status

	Public-sector patients with HIV			Public-sector patients without HIV		
	No diagnosed COVID-19, n = 536 574	COVID-19 cases, not deceased, n = 3863	COVID-19 cases, deceased, n = 115	No diagnosed COVID-19, n = 2 902 050	COVID-19 cases, not deceased, n = 17 820	COVID-19 cases, deceased, n = 510
Sex						
Female	356 356 (66%)	3039 (79%)	62 (54%)	1 627 124 (56%)	11 877 (67%)	278 (55%)
Male	180 218 (34%)	824 (21%)	53 (46%)	1 274 926 (44%)	5943 (34%)	232 (45%)
Age						
20–39 years	310 551 (58%)	2187 (57%)	17 (15%)	1 603 235 (55%)	9453 (53%)	29 (6%)
40–49 years	147 344 (27%)	1136 (29%)	28 (24%)	457 632 (16%)	3379 (19%)	35 (7%)
50–59 years	59 345 (11%)	418 (11%)	40 (35%)	388 394 (13%)	2809 (16%)	122 (24%)
60–69 years	15 856 (3%)	98 (3%)	21 (18%)	260 226 (9%)	1325 (7%)	157 (31%)
≥70 years	3473 (1%)	24 (1%)	9 (8%)	192 562 (7%)	854 (5%)	167 (33%)
Diabetes						
None	517 609 (96%)	3491 (90%)	57 (50%)	2 659 479 (92%)	15 090 (85%)	196 (38%)
Diabetes HbA1c <7%	3493 (1%)	65 (2%)	8 (7%)	41 561 (1%)	426 (2%)	50 (10%)
Diabetes HbA1c 7–8.9%	2998 (1%)	77 (2%)	16 (14%)	44 213 (2%)	505 (3%)	78 (15%)
Diabetes HbA1c ≥9%	4562 (1%)	126 (3%)	25 (22%)	61 077 (2%)	960 (5%)	133 (26%)
Diabetes, no HbA1c measurement	7912 (1%)	104 (3%)	9 (8%)	95 720 (3%)	839 (5%)	53 (10%)
Other noncommunicable diseases						
Hypertension	62 676 (12%)	692 (18%)	48 (42%)	501 232 (18%)	4218 (24%)	314 (62%)
Chronic kidney disease	6348 (1%)	82 (2%)	21 (18%)	55 319 (2%)	412 (2%)	90 (18%)
Chronic pulmonary disease/asthma	23 501 (4%)	218 (6%)	10 (9%)	169 086 (6%)	1359 (8%)	74 (15%)
Tuberculosis						
Previous tuberculosis	129 259 (24%)	864 (22%)	42 (37%)	157 630 (5%)	834 (5%)	45 (9%)
Current tuberculosis	24 357 (5%)	172 (4%)	16 (14%)	29 895 (1%)	145 (1%)	10 (2%)

Data are of Western Cape active patients ≥20 years of age in the public sector (public-sector health-care visit in last 3 years) with and without HIV according to COVID-19 outcome. Column percentages may add up to >100% due to rounding.

Abbreviations: COVID-19, coronavirus disease 2019; HbA1c, glycosylated haemoglobin; HIV, human immunodeficiency virus.

### COVID-19 Death in All Public-sector Patients

Among all public-sector patients, the probability of COVID-19 death by 100 days after 1 March 2020 was 180/million (95% CI, 167–196). COVID-19 death was associated with male sex, increasing age, diabetes (with a higher risk with elevated HbA1c), hypertension, and chronic kidney disease (Table 3). Current tuberculosis was associated with an increased hazard of COVID-19 death (adjusted hazard ratio [aHR], 2.70; 95% CI, 1.81–4.04), with a smaller increase for having previous tuberculosis (aHR, 1.51; 95% CI, 1.18–1.93). The increased hazard of COVID-19 death associated with current tuberculosis was present for both microbiologically confirmed and unconfirmed tuberculosis and for rifampicin-sensitive and -resistant disease during intensive phase treatment (Supplementary Table 3).

**Table 3.** Associations with Coronavirus Disease 2019 Death Among All Public-sector Patients ≥20 Years Old With a Public-sector Health Visit in the Previous 3 Years

	Adjusted for location only			Adjusted for age and sex			Adjusted for all variables listed		
	HR	95% CI	*P* value	Adjusted HR	95% CI	*P* value	Adjusted HR	95% CI	*P* value
Sex									
Female	Ref	…		Ref	…		Ref	…	
Male	1.21	1.03–1.41	.02	1.26	1.07–1.47	.005	1.45	1.23–1.70	<.001
Age									
20–39 years	Ref	…		Ref	…		Ref	…	
40–49 years	4.46	3.05–6.52	<.001	4.42	3.02–6.46	<.001	2.83	1.92–4.15	<.001
50–59 years	16.23	11.70–22.52	<.001	16.13	11.62–22.39	<.001	7.78	5.51–10.98	<.001
60–69 years	28.82	20.83–39.87	<.001	28.81	20.82–39.86	<.001	11.54	8.11–16.42	<.001
≥70 years	41.37	29.87–57.29	<.001	41.85	30.21–57.96	<.001	16.79	11.69–24.11	<.001
Diabetes									
None	Ref	…		Ref	…		Ref	…	
Diabetes HbA1c <7%	16.59	12.47–22.09	<.001	6.07	4.52–8.16	<.001	5.37	3.96–7.27	<.001
Diabetes HbA1c 7–8.9%	25.32	19.98–32.10	<.001	9.26	7.23–11.85	<.001	8.53	6.60–11.02	<.001
Diabetes HbA1c ≥9%	29.57	24.23–36.10	<.001	12.90	10.47–15.88	<.001	12.07	9.70–15.02	<.001
Diabetes, no HbA1c measurement	7.29	5.52–9.62	<.001	3.02	2.27–4.02	<.001	2.91	2.18–3.89	<.001
Other noncommunicable diseases									
Hypertension	6.72	5.73–7.88	<.001	2.20	1.85–2.62	<.001	1.31	1.09–1.57	.004
Chronic kidney disease	11.43	9.30–14.05	<.001	3.21	2.57–4.01	<.001	1.86	1.49–2.33	<.001
Chronic pulmonary disease / asthma	2.49	1.98–3.13	<.001	1.08	.85–1.36	.538	.93	.73–1.17	.514
Tuberculosis									
Never tuberculosis	Ref	…		Ref	…		Ref	…	
Previous tuberculosis	1.79	1.42–2.24	<.001	1.81	1.44–2.28	<.001	1.51	1.18–1.93	.001
Current tuberculosis	2.79	1.88–4.13	<.001	3.29	2.21–4.88	<.001	2.70	1.81–4.04	<.001
HIV									
Negative	Ref	…		Ref	…		Ref	…	
Positive	1.07	.88–1.32	.494	1.97	1.59–2.45	<.001	2.14	1.70–2.70	<.001
VL <1000 copies/ml (last 15 mo) and ART script (last 6 mo)^a^	…	…		…	…		2.61	1.98–3.43	<.001
VL <1000 copies/ml (2yr to 15 mo prior), or ART script (last 6 mo) and VL <1000 copies/ml >2yr prior	…	…		…	…		1.76	.96–3.24	.067
VL ≥1000 copies/ml (last 15 mo) or CD4 count <200 cells/µl (last 18 mo)	…	…		…	…		3.35	1.83–6.12	<.001
No VL (last 15 mo); CD4 count ≥200 cells/µl or unknown (last 18 mo)	…	…		…	…		1.33	.85–2.07	.217

Data are from univariate and multivariate HRs and 95% CIs from 1 March to 9 June 2020, for patients (n = 3 460 932), using Cox proportional hazards models.

Abbreviations: ART, antiretroviral therapy; CI, confidence interval; HbA1c, glycosylated haemoglobin; HIV, human immunodeficiency virus; HR, hazard ratio; VL, viral load.

^a^Reference category for HR is HIV negative, only included in an adjusted analysis, adjusted for all other variables listed in this table in a model that included the listed categories of HIV VL, ART, and immunosuppression instead of the binary variable of testing HIV positive vs negative; the effect of the other variables on mortality was similar to those presented here.

After adjusting for age, sex, and other comorbidities, HIV was associated with increased COVID-19 mortality (aHR, 2.14; 95% CI, 1.70–2.70), and this association was similar irrespective of viremia or immunosuppression prior to the COVID-19 episode. However, few patients were viremic or immunosuppressed, as reflected in the wide CIs for the hazard ratios in different groups. Associations with most comorbidities increased when restricting to those with high-certainty comorbidity evidence (Supplementary Table 4). The associations of most comorbidities with COVID-19 death were attenuated when restricting to patients with ≥1 medical visit/year in the last 3 years, as these patients were more likely to have comorbidities warranting regular visits; however, HIV remained significantly associated with COVID-19 death (Supplementary Table 4). Among all public-sector adults, 9.8% of COVID-19 deaths were attributable to HIV (95% CI, 6.2–13.3), 2.6% (95% CI, 1.0–4.2) to current tuberculosis, and 4.7% (95% CI, 1.5–7.8) to previous tuberculosis.

### Death in COVID-19 Cases and Hospitalized Patients

**Figure 1.**
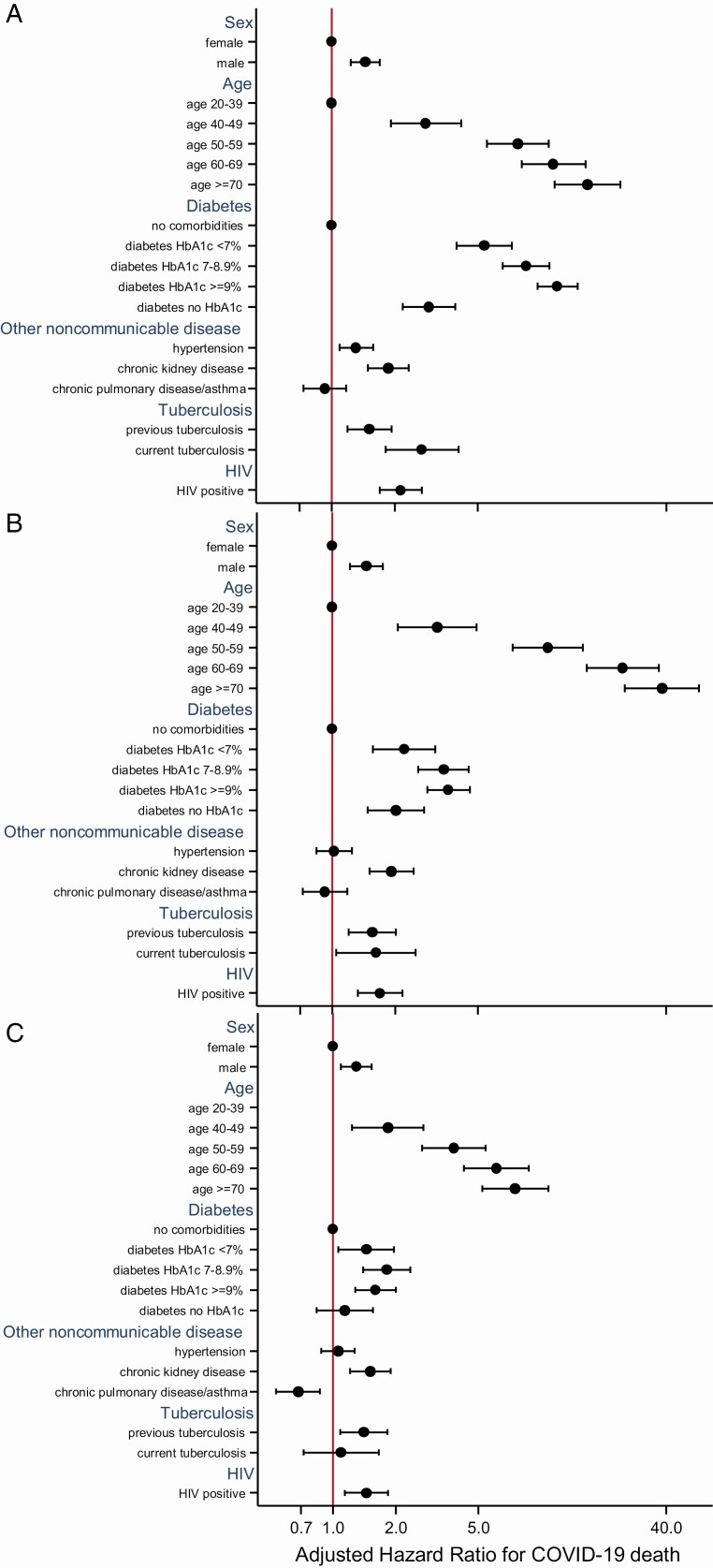
Comparison of adjusted HRs and 95% CIs for associations with COVID-19 death from Cox proportional hazards models among (*A*) all public-sector patients ≥20 years old with a public-sector health visit in the previous 3 years (n = 3 460 932); (*B*) all adult COVID-19 cases diagnosed before 1 June 2020 (n = 15 203); and (*C*) all hospitalized COVID-19 cases (n = 2978). Abbreviations: CI, confidence interval; COVID-19, coronavirus disease 2019; HbA1c, glycosylated hemoglobin; HIV, human immunodeficiency virus; HR, hazard ratio.

Among 15 203 COVID-19 cases diagnosed before 1 June 2020, the associations of all comorbidities with COVID-19 death were attenuated compared to the population analysis. However, HIV (aHR, 1.70; 95% CI, 1.32–2.18), current tuberculosis (aHR, 1.62; 95% CI, 1.04–2.51), and previous tuberculosis (aHR, 1.55; 95% CI, 1.19–2.02) remained associated with COVID-19 death (Table 4; Figure 1). Results were similar when including patients diagnosed after the change in testing criteria (Supplementary Table 5). Among COVID-19 cases in PLWH on ART, mortality was lower in patients on TDF (vs abacavir/zidovudine; aHR, 0.41; 95% CI, .21–.78), with no differences in mortality rates for other antiretrovirals.

**Table 4.** Associations with Coronavirus Disease 2019 Death from Cox Proportional Hazards Models

	All public-sector SARS-CoV-2 cases diagnosed before 1 June 2020,^a^ n = 15 203			Hospitalized public-sector SARS-CoV-2 cases, n = 2978		
	Adjusted HR	95% CI	*P* value	Adjusted HR	95% CI	*P* value
Sex						
Female	Ref	…		Ref	…	
Male	1.45	1.22–1.74	<.001	1.29	1.09–1.53	.003
Age						
20–39 years	Ref	…		Ref	…	
40–49 years	3.19	2.06–4.93	<.001	1.83	1.23–2.72	.003
50–59 years	10.84	7.34–16.01	<.001	3.81	2.68–5.42	<.001
60–69 years	24.87	16.67–37.11	<.001	6.11	4.27–8.75	<.001
≥70 years	38.32	25.47–57.64	<.001	7.53	5.23–10.84	<.001
Diabetes						
None	Ref	…		Ref	…	
Diabetes HbA1c <7%	2.21	1.57–3.12	<.001	1.44	1.06–1.96	.020
Diabetes HbA1c 7–8.9%	3.41	2.59–4.51	<.001	1.81	1.39–2.35	<.001
Diabetes HbA1c ≥9%	3.62	2.85–4.59	<.001	1.60	1.27–2.0	<.001
Diabetes, no HbA1c measurement	2.02	1.47–2.76	<.001	1.13	.83–1.55	<.001
Other noncommunicable diseases						
Hypertension	1.02	.84–1.24	.843	1.05	.88–1.27	.574
Chronic kidney disease	1.92	1.51–2.45	<.001	1.51	1.20–1.89	<.001
Chronic pulmonary disease/asthma	.92	.72–1.18	.512	.68	.53–.86	.002
Tuberculosis						
Never tuberculosis	Ref	…		Ref	…	
Previous tuberculosis	1.55	1.19–2.02	.001	1.40	1.08–1.82	.011
Current tuberculosis	1.62	1.04–2.51	.031	1.09	.72–1.65	.683
HIV						
Negative	Ref	…		Ref	…	
Positive	1.70	1.32–2.18	<.001	1.45	1.14–1.84	.002
VL <1000 copies/ml (last 15 mo) and ART script (last 6 mo)^b^	1.60	1.19–2.17	.002	1.57	1.18–2.07	.002
VL <1000 copies/ml (2yr to 15 mo prior), or ART script (last 6 mo) and VL <1000 copies/ml >2yr prior	1.56	.80–3.07	.193	1.33	.72–2.46	.357
VL ≥1000 copies/ml (last 15 mo) or CD4 count <200 cells/µl (last 18 mo)	3.39	1.14–3.62	<.001	1.60	.79–3.25	.190
No VL (last 15 mo); CD4 count ≥200 cells/µl or unknown (last 18 mo)	1.73	1.10–2.71	.017	1.17	.73–1.87	.506
ART in PLWH with script issued in last 12 months^c^						
Abacavir or zidovudine	Ref	…		Ref	…	
Tenofovir disoproxil fumarate	.41	.21–.78	.007	.57	.31–1.04	.067
Efavirenz	Ref	…		Ref	…	
Lopinavir	.91	.37–2.25	.846	.68	.29–1.63	.392
Atazanavir	.38	.05–2.92	.352	1.09	.25–4.82	.911
Dolutegravir	.57	.16–2.01	.380	.62	.17–2.22	.461
ART duration						
<1 year	Ref	…		Ref	…	
1–2 years	.78	.21–2.94	.719	1.28	.37–4.42	.701
≥2 years	.54	.19–1.48	.230	.55	.21–1.42	.213
CD4 count during COVID-19^d^						
>350 cells/µl	…	…		1.24	.95–1.63	.112
200–349 cells/µl	…	…		1.65	.94–2.88	.080
<200 cells/µl	…	…		2.36	1.47–3.78	<.001

Data are multivariate HRs and 95% CIs among (1) all adult COVID-19 cases diagnosed before 1 June 2020 (n = 15 203) and (2) all hospitalized adult COVID-19 cases (n = 2978).

Abbreviations: ART, antiretroviral therapy; CI, confidence interval; COVID-19, coronavirus disease 2019; HbA1c, glycosylated haemoglobin; HIV, human immunodeficiency virus; HR, hazard ratio; PLWH, people living with HIV; SARS-CoV-2, severe acute respiratory syndrome coronavirus 2; VL, viral load.

^a^Analysis limited to cases diagnosed before 1 June 2020, when testing criteria changed, with public-sector tests being limited to patients >55 year of age or with comorbidities.

^b^Reference category is HIV negative; adjusted for all other variables listed in this table in a model that included the listed categories of HIV VL, CD4 count, and ART instead of the binary variable of testing HIV positive vs negative; the effect of the other variables on mortality was similar to those presented here.

^c^Restricted to patients with documented antiretrovirals dispensed in the last 12 months, adjusted for all other variables listed in this table in a model that included the relevant antiretrovirals and ART duration; the effect of the other variables on mortality was similar to those presented here.

^d^Reference category is HIV negative, restricted to patients living without HIV and 199 of 601 PLWH with a CD4 count measurement at the time of COVID-19 diagnosis or admission and adjusted for all other variables listed in this table in a model that included the listed categories of CD4 count instead of the binary variable of testing HIV positive vs negative; the effect of the other variables on mortality was similar to those presented here.

Among hospitalized COVID-19 cases, the associations of all mortality risk factors, including HIV, were attenuated, but HIV remained associated with death (aHR, 1.45; 95% CI, 1.14–1.84), as did previous tuberculosis (aHR, 1.40; 95% CI, 1.08–1.82), but not current tuberculosis. Among 601 hospitalized PLWH, 199 (33%) had their CD4 count measured during the COVID-19 episode, of whom 70 (35%) had CD4 count <200 cells/µl, which was associated with COVID-19 death (aHR vs people living without HIV, 2.36 [95% CI, 1.47–3.78]; aHR vs PLWH with CD4 count ≥350 cells/µl, 1.97 [95% CI, 1.14–3.40]). Among the 70 PLWH with a CD4 count < 200 cells/µl during admission, 4 (5%) had no prior evidence of HIV in the WCPHDC; 47% had a previous CD4 count <200 cells/µl, an unsuppressed viral load, or no recent ART; and 47% had a previous CD4 count ≥200 cells/µl or appeared stable on ART (viral load [VL] <1000 copies/ml or ART dispensed in last year; Supplementary Figure 1).

### Potential Bias from Unmeasured Confounding

To assess whether the association between HIV or tuberculosis and COVID-19 mortality could be due to residual unmeasured confounding—for example, by socioeconomic status or unrecorded comorbidities—we calculated the E-value for an unmeasured confounder. For HIV, the E-value for the analysis among all public-sector patients was 3.70 (and 2.79 for the lower bound of the CI), suggesting that only a strong association between HIV and a confounder (eg, socioeconomic status) and between the confounder and COVID-19 death would account for all of the observed association between HIV and COVID-19 death. The effect of HIV on COVID-19 death was similar when restricted to the poorest subdistrict, with the highest HIV prevalence, in Cape Town [19]. Corresponding E-values for current and previous tuberculosis were 4.84 (3.02 for the lower bound of CI) and 2.39 (1.64 for the lower bound of CI), respectively. A quantitative bias analysis showed that the HIV-associated increased risk of COVID-19 death was unlikely due to confounding by raised BMI (Supplementary Appendix 4).

### Standardized Mortality Ratio

Among all laboratory-diagnosed COVID-19 cases, there were 135 deaths among an estimated ~520 000 PLWH in the province (260 deaths/million) and 786 deaths among 6.36 million people without HIV (124 deaths/million). The SMR for COVID-19 mortality in PLWH, relative to people living without HIV, was 2.39 (95% CI, 1.96–2.86), and the attributable fraction of public- and private-sector COVID-19 deaths due to HIV was 8.5% (95% CI, 6.1–11.1).

## DISCUSSION

Among nearly 3.5 million adults (16% PLWH) in South Africa, we found an approximately 2-fold association of COVID-19 death with HIV, irrespective of viremia or immunosuppression prior to the COVID-19 episode, and a similar association between COVID-19 death and current tuberculosis. Among PLWH on ART, receiving TDF was associated with lower COVID-19 mortality, compared to receipt of other antiretrovirals. While the HIV- and tuberculosis-associated increased risks of COVID-19 death may be overestimated if there is residual confounding, due to socioeconomic status or unrecorded comorbidities, our results, supported by sensitivity analyses, demonstrate that PLWH and persons with tuberculosis are at increased risk of severe COVID-19. Nonetheless, despite a high burden of advanced HIV in the province, the attributable fraction of all deaths ascribed to HIV was <10%.

While most case series of HIV and SARS-CoV-2 coinfection have not shown poor outcomes in PLWH [1–3, 5–7], some cohorts of hospitalized PLWH with COVID-19 have reported substantial morbidity and mortality, including among patients with suppressed VLs on ART [23, 24]. Comparisons by HIV status of hospitalized COVID-19 cases in New York and London have not shown differences in mortality risks [25–27]. However, the absence of an increased mortality risk in hospitalized patients with comorbidities may be explained by selection bias: risk factors for COVID-19 death may be attenuated by restricting data to the subset of hospitalized patients already at high mortality risk [28]. It is therefore expected that the increased risk of death associated with all comorbidities in our analysis was progressively attenuated when restricting to cases (people with sufficiently severe symptoms to be tested) and hospitalized patients.

Similar to our findings, several studies have reported a high prevalence of comorbidities among PLWH with severe COVID-19 [3, 6, 7]. The high prevalence of comorbidities in deceased PLWH suggests that the effect of HIV may at least partly be due to an increased risk of comorbidities at younger ages [2, 7], including those not recorded in WCPHDC, such as cardiovascular disease. Persistent immune dysfunction may also be important in severe COVID-19 despite viral suppression; the hazard ratio point estimates for association with COVID-19 death were greater in immunosuppressed or viremic PLWH, although the numbers of these patients with COVID-19 were small, with wide CIs. Further, a CD4 count < 200 cells/µl during admission was associated with COVID-19 death. While this may partly be due to the well-described lymphopenia in severe COVID-19, which is prognostic of poor outcomes, about half of patients with low CD4 counts during admission were either new HIV diagnoses or had previous immunosuppression, viremia, or no recent ART [10]. Among COVID-19 cases in PLWH on ART, the receipt of TDF (vs other therapies) was associated with reduced COVID-19 mortality. However, this association is likely to be overestimated; in South Africa, only patients on second-line ART or with poor renal function would not be on TDF, and both of these factors may themselves increase mortality. Nonetheless, the association remained when adjusting for kidney disease, viral suppression, and ART duration, and concurs with results from a recently published cohort of PLWH on ART from Spain [13]. We found both current and previous tuberculosis to be associated with COVID-19 death, but since current tuberculosis itself causes death, in the absence of autopsy evidence it is difficult to disentangle the effects of COVID-19 versus tuberculosis disease on mortality [17].

In our study, the overall high prevalence of diabetes in people with and without HIV, high proportion with poor glycemic control, and very elevated risks for COVID-19 death for diabetics, compared to data reported from other countries, are concerning [8]. Diabetes is often diagnosed late and/or untreated or poorly controlled in resource-limited settings, and the resulting microvascular disease, even in people with good current diabetic control, may increase COVID-19 mortality [29].

To our knowledge, this is the largest report on SARS-CoV-2 from Africa, the largest report on HIV and tuberculosis coinfected patients, and the first comparison of COVID-19 outcomes in patients with and without tuberculosis. Strengths include the study size using population-level data, laboratory-confirmed SARS-CoV-2 diagnoses in all COVID-19 cases, and the inclusion of hospitalized and nonhospitalized cases and deaths, as well as modeling the independent associations of HIV and tuberculosis with COVID-19 death. While the population analysis approach is robust to selection bias associated with cases and hospitalized patients only, it may overestimate associations between comorbidities and COVID-19 death if those with comorbidities live in areas with higher transmission or have closer follow-up and are more likely to be diagnosed with COVID-19. Nonetheless the coherence of associations found when analyzing the population cohort and SMR, diagnosed cases and hospitalized patients suggest that the population findings are unlikelyto be solely due to different probabilities of encountering SARS-CoV2 or being diagnosed once infected. Furthermore, an adjustment for subdistrict of residence should address geographic differences in transmission probability. Being an observational study, limitations include the underascertainment of comorbidities in routine administrative data; a lack of data on other potential risk factors, including socioeconomic status, smoking, and BMI; possible underascertainment of all COVID-19 cases and deaths; and potential misclassification of some incidental deaths in patients positive for SARS-CoV-2 as related to COVID-19, although almost all deaths occurred in hospitalized patients with clinical COVID-19. Further, we were unable to systematically exclude other opportunistic infections as contributors to COVID-19 mortality, as investigation for these causes varied by facility, clinical presentation, and time in hospital. Relatively large numbers of PLWH had no recent VL or CD4 count results, limiting our ability to distinguish outcomes for different strata of these measures. In particular, patients with no recent information on disease control (eg, HIV RNA or HBA1c) may have less contact with health services and not reside permanently in the province, with underascertainment of outcomes.

## CONCLUSION

While our findings of increased COVID-19 mortality risks in those living with HIV or tuberculosis may overestimate associations of these conditions with COVID-19 death due to residual confounding, PLWH and/or those with tuberculosis should nonetheless be considered high-risk groups for COVID-19 management, especially if they have other comorbidities.

## Supplementary Data

Supplementary materials are available at *Clinical Infectious Diseases* online. Consisting of data provided by the authors to benefit the reader, the posted materials are not copyedited and are the sole responsibility of the authors, so questions or comments should be addressed to the corresponding author.

ciaa1198_suppl_Supplementary_Material

## Notes

List of Contributing Authors

Western Cape Government: Health

*Health Impact Assessment Directorate:* Andrew Boulle,^1,2^ Mary-Ann Davies,^1,2^ Hannah Hussey,^1,3^ Muzzammil Ismail,^1,3^ Erna Morden,^1,3^ Ziyanda Vundle,^1,4^ Virginia Zweigenthal,^1,3^*Metro and Rural Health Outbreak Response Team Leads*: Hassan Mahomed,^4,5^ Masudah Paleker,^4,5^ David Pienaar,^6^ Yamanya Tembo^3,6^*Communicable Disease Control sub-Directorate*: Charlene Lawrence,^7^ Washiefa Isaacs,^7^ Hlengani Mathema^7,8^*Provincial Health Data Centre:* Derick Allen,^2^ Taryn Allie,^1,2^ Jamy-Lee Bam,^1^ Kasturi Buddiga,^1,2^ Pierre Dane,^1,2^ Alexa Heekes,^1,2^ Boitumelo Matlapeng,^1,2^ Themba Mutemaringa,^1,2^ Luckmore Muzarabani,^1,2^ Florence Phelanyane,^1,2^ Rory Pienaar,^1^ Catherine Rode,^1,2^ Mariette Smith,^1,2^ Nicki Tiffin,^1,2,9,10^ Nesbert Zinyakatira^1,3^*Health Programmes Directorate*: Carol Cragg,^11^ Frederick Marais,^11,12^ Vanessa Mudaly,^3,11^ Jacqueline Voget^11^*Hospitals: George Hospital*: Jody Davids,^13^ Francois Roodt,^13^ Nellis van Zyl Smit,^13^ Alda Vermeulen^13^*Groote Schuur Hospital*: Kevin Adams,^14,15^ Gordon Audley,^14,16^ Kathleen Bateman,^14,16^ Peter Beckwith,^14,16^ Marc Bernon,^14,15^ Dirk Blom,^14,16^ Linda Boloko,^14,16^ Jean Botha,^14,16^ Adam Boutall,^14,15^ Sean Burmeister,^14,15^ Lydia Cairncross,^14,15^ Gregory Calligaro,^14,16^ Cecilia Coccia,^14,16^ Chadwin Corin,^14,16^ Remy Daroowala,^14,15^ Joel A Dave,^14,16^ Elsa De Bruyn,^14,16^ Martin De Villiers,^14,16^ Mimi Deetlefs,^14,16^ Sipho Dlamini,^14,16^ Thomas Du Toit,^14,16^ Wilhelm Endres,^14,16^ Tarin Europa,^14,16^ Graham Fieggan,^14,15^ Anthony Figaji,^14,15^ Petro Frankenfeld,^14,16^ Elizabeth Gatley,^14,16^ Phindile Gina,^14,16^ Evashan Govender,^14,16^ Rochelle Grobler,^14,16^ Manqoba Vusumuzi Gule,^14,16^ Christoff Hanekom,^14,16^ Michael Held,^14,16^ Alana Heynes,^14,16^ Sabelo Hlatswayo,^14,16^ Bridget Hodkinson,^14,16^ Jeanette Holtzhausen,^14^ Shakeel Hoosain,^14,16^ Ashely Jacobs,^14,16^ Miriam Kahn,^14,15^ Thania Kahn,^14,16^ Arvin Khamajeet,^14,15^ Joubin Khan,^14,16^ Riaasat Khan^14,16^ Alicia Khwitshana,^14,16^ Lauren Knight,^14,16^ Sharita Kooverjee,^14,16^ Rene Krogscheepers,^14,16^ Jean Jacque Kruger,^14,16^ Suzanne Kuhn,^14,16^ Kim Laubscher,^14,15^ John Lazarus,^14,15^ Jacque Le Roux,^14,16^ Scott Lee Jones,^14,16^ Dion Levin,^14,16^ Gary Maartens,^14,16^ Thina Majola,^14,16^ Rodgers Manganyi,^14,16^ David Marais,^14,16^ Suzaan Marais,^14,16^ Francois Maritz,^14,15^ Deborah Maughan,^14,16^ Simthandile Mazondwa,^14,16^ Luyanda Mbanga,^14,16^ Nomonde Mbatani,^14,16^ Bulewa Mbena,^14,16^ Graeme Meintjes,^14,16^ Marc Mendelson,^14,16^ Ernst Möller,^14,16^ Allison Moore,^14,15^ Babalwa Ndebele,^14,16^ Marc Nortje,^14,15^ Ntobeko Ntusi,^14,16^ Funeka Nyengane,^14,16^ Chima Ofoegbu,^14,15^ Nectarios Papavarnavas,^14,16^ Jonny Peter,^14,16^ Henri Pickard,^14,15^ Kent Pluke,^14,15^ Peter J Raubenheimer,^14,16^ Gordon Robertson,^14,16^ Julius Rozmiarek,^14,16^ A Sayed,^14,16^ Matthias Scriba,^14,15^ Hennie Sekhukhune,^14,16^ Prasun Singh,^14,16^ Elsabe Smith,^14,16^ Vuyolwethu Soldati,^14,15^ Cari Stek,^14,16^ Robert van den berg,^14,16^ Le Roux van der Merwe,^14,16^ Pieter Venter,^14,15^ Barbra Vermooten,^14,16^ Gerrit Viljoen,^14,16^ Santhuri Viranna,^14,16^ Jonno Vogel,^14,16^ Nokubonga Vundla,^14,16^ Sean Wasserman,^14,16^ Eddy Zitha^14,16^*Helderberg Hospital*: Vanessa Lomas-Marais,^17^ Annie Lombard,^17^ Katrin Stuve,^17^ Werner Viljoen^17^*Karl Bremer Hospital*: De Vries Basson,^18^ Sue Le Roux,^18^ Ethel Linden-Mars,^18^ Lizanne Victor,^18^ Mark Wates,^18^ Elbe Zwanepoel^18^*Khayelitsha District Hospital*: Nabilah Ebrahim,^19^ Sa’ad Lahri,^19^ Ayanda Mnguni^19^*Mitchells Plain Hospital*: Thomas Crede,^20^ Martin de Man,^20,31^ Katya Evans,^20,31^ Clint Hendrikse,^20,31^ Jonathan Naude,^20^ Moosa Parak,^20,31^ Patrick Szymanski,^20^ Candice Van Koningsbruggen^20,31^*Tygerberg Hospital*: Riezaah Abrahams,^21,22^ Brian Allwood,^21,22^ Christoffel Botha,^21,22^ Matthys Henndrik Botha,^21,23^ Alistair Broadhurst,^21,22^ Dirkie Claasen,^21,22^ Che Daniel,^21,22^ Riyaadh Dawood,^21,22^ Marie du Preez,^21,22^ Nicolene Du Toit,^21,23^ Kobie Erasmus,^21,24^ Coenraad F N Koegelenberg,^21,22^ Shiraaz Gabriel,^21,22^ Susan Hugo,^21,22^ Thabiet Jardine,^21,22^ Clint Johannes,^21,22^ Sumanth Karamchand,^21,22^ Usha Lalla,^21,22^ Eduard Langenegger,^21,23^ Eize Louw,^21,22^ Boitumelo Mashigo,^21,22^ Nonte Mhlana,^21,22^ Chizama Mnqwazi,^21,22^ Ashley Moodley,^21,23^ Desiree Moodley,^21,22^ Saadiq Moolla,^21,22^ Abdurasiet Mowlana,^21,22^ Andre Nortje,^21,22^ Elzanne Olivier,^21,23^ Arifa Parker,^21,22^ Chané Paulsen,^21,22^ Hans Prozesky,^21,22^ Jacques Rood,^21,22^ Tholakele Sabela,^21,22^ Neshaad Schrueder,^21,22^ Nokwanda Sithole,^21,22^ Sthembiso Sithole,^21,22^ Jantjie J Taljaard,^21,22^ Gideon Titus,^21,22^ Tian Van Der Merwe,^21,23^ Marije van Schalkwyk,^21,22^ Luthando Vazi,^21,22^ Abraham J Viljoen,^21,22^ Mogamat Yazied Chothia^21,22^*Emergency Medical Services*: Vanessa Naidoo,^24^ Lee Alan Wallis^24,31^*District Outbreak Response Teams:* Mumtaz Abbass,^25^ Juanita Arendse,^25^ Rizqa Armien,^25^ Rochelle Bailey,^25^ Muideen Bello,^25^ Rachel Carelse,^25^ Sheron Forgus,^25^ Nosi Kalawe,^25^ Saadiq Kariem,^25^ Mariska Kotze,^25^ Jonathan Lucas,^25^ Juanita McClaughlin,^25^ Kathleen Murie,^25^ Leilah Najjaar,^25^ Liesel Petersen,^25^ James Porter,^25^ Melanie Shaw,^25^ Dusica Stapar,^25^ Michelle Williams^25^

**City of Cape Town:** Linda Aldum,^26^ Natacha Berkowitz,^26^ Raakhee Girran,^26^ Kevin Lee,^26^ Lenny Naidoo,^26^ Caroline Neumuller^26^

**Outbreak Response Pod Team:** Kim Anderson,^2^ Kerrin Begg,^3^ Lisa Boerlage,^3^ Morna Cornell,^2^ Renée de Waal,^2^ Lilian Dudley,^5^ René English,^5^ Jonathan Euvrard,^2^ Pam Groenewald,^27^ Nisha Jacob,^3^ Heather Jaspan,^32^ Emma Kalk,^2^ Naomi Levitt,^16^ Thoko Malaba,^3^ Patience Nyakato,^2^ Gabriela Patten,^2^ Helen Schneider,^28^ Maylene Shung King,^3^ Priscilla Tsondai,^2^ James Van Duuren,^3^ Nienke van Schaik

**National Institute for Communicable Diseases**: Lucille Blumberg,^8,33^ Cheryl Cohen,^8,34^ Nelesh Govender,^8,29^ Waasila Jassat,^8^ Tendesayi Kufa,^8^ Kerrigan McCarthy,^8^ Lynn Morris^8,35^

**National Health Laboratory Service**: Nei-yuan Hsiao,^30^ Ruan Marais^30^

**Statistical and Analytic Support**: Jon Ambler,^9^ Olina Ngwenya,^9^ Richard Osei-Yeboah,^10^ Leigh Johnson,^2^ Reshma Kassanjee,^2^ Tsaone Tamuhla^10^

**Affiliations**

^1^Health Impact Assessment, Western Cape Government: Health

^2^Centre for Infectious Disease Epidemiology and Research, School of Public Health and Family Medicine, University of Cape Town

^3^School of Public Health and Family Medicine, University of Cape Town

^4^Division of Health Systems and Public Health, Department of Global Health, Faculty of Medicine and Health Sciences, Stellenbosch University

^5^Metro Health Services, Western Cape Government: Health

^6^Rural Health Services, Western Cape Government: Health

^7^Communicable Disease Sub-Directorate, Western Cape Government: Health

^8^National Institute for Communicable Diseases, National Health Laboratory Service, South Africa

^9^Wellcome Centre for Infectious Disease Research in Africa, University of Cape Town

^10^Division of Computational Biology, University of Cape Town

^11^Health Programmes Directorate, Western Cape Government: Health

^12^Faculty of Health Sciences, North West University

^13^George Hospital, Western Cape Government: Health

^14^Groote Schuur Hospital, Western Cape Government: Health

^15^Department of Surgery, University of Cape Town

^16^Department of Medicine, University of Cape Town

^17^Department of Radiology, University of Cape Town

^18^Karl Bremer Hospital, Western Cape Government: Health

^19^Khayelitsha District Hospital, Western Cape Government: Health

^20^Mitchells Plain and Heideveld Hospitals, Western Cape Government: Health

^21^Tygerberg Hospital, Western Cape Government: Health

^22^Department of Medicine, Stellenbosch University

^23^Department of Obstetrics and Gyneacology, Stellenbosch University

^24^Emergency Medical Services, Western Cape Government

^25^Western Cape Government: Health

^26^City Health, Community Services and Health, City of Cape Town

^27^South African Medical Research Council Burden of Disease Research Unit

^28^School of Public Health, University of the Western Cape

^29^School of Pathology, University of the Witwatersrand and School of Pathology, University of Cape Town

^30^National Health Laboratory Service and Division of Virology, School of Pathology, University of Cape Town

^31^Division of Emergency Medicine, University of Cape Town

^32^Division of Immunology and Institute of Infectious Diseases and Molecular Medicine, University of Cape Town

^33^University of Pretoria

^34^School of Public Health, University of Witwatersrand

^35^University of Witwatersrand, South African Medical Research Council Antibody Immunity Research Unit and the Centre for the AIDS Programme in South Africa (CAPRISA)

***Acknowledgments.*** The author thanks all patients in the Western Cape and the Western Cape Department of Health Provincial Health Data Centre, the Western Cape Department of Health Coronavirus Disease 2019 (COVID-19) Outbreak Response Team, the Western Cape Communicable Disease Control sub-directorate, and Western Cape health-care workers involved in the COVID-19 response for their contributions to this report.

***Financial support.*** This work was supported by the Western Cape Provincial Health Data Centre from the Western Cape Department of Health, the US National Institutes for Health (grant numbers R01 HD080465 and U01 AI069924), the Bill and Melinda Gates Foundation (grant numbers 1164272 and 119327), the United States Agency for International Development (grant number 72067418CA00023), and the Wellcome Trust (grant number 203135/Z/16/Z).

***Potential conflict of interest.*** M.-A. D. reports grants from Viiv Healthcare, outside the submitted work. A. B. reports grants to their institution from National Institutes of Health, Bill and Melinda Gates Foundation, and Wellcome Trust; and nonfinancial support from United States Agency for International Development. All authors have submitted the ICMJE Form for Disclosure of Potential Conflicts of Interest. Conflicts that the editors consider relevant to the content of the manuscript have been disclosed.

All contributing authors are listed at the end of the paper.
