# Supplementary material for: Risk Factors for Coronavirus Disease 2019 (COVID-19) Death in a Population Cohort Study from the Western Cape Province, South Africa
Source: Clin Infect Dis. 2020 Aug 29;73(7):e2005–15. doi: 10.1093/cid/ciaa1198 (PMC7499501; doi:10.1093/cid/ciaa1198)
Supplement: ciaa1198_suppl_Supplementary_Material [file ciaa1198_suppl_supplementary_material.docx]

**Supplementary Appendix**

**Risk factors for COVID-19 death in a population cohort study from
the Western Cape Province, South Africa.**

Table of Contents

[**1.** **Enumeration of different health conditions from the Western Cape Provincial Health Data Centre** 2](#_Toc46994517)

[**Supplementary Table 1** 3](#_Toc46994518)

[**2.** **Calculation of the Standardized Mortality Ratios (SMR) and population attributable fractions** 4](#_Toc46994519)

[**3.** **Supplementary Results** 6](#_Toc46994520)

[**Supplementary Table 2** 6](#_Toc46994521)

[**Supplementary Table 3a** 7](#_Toc46994522)

[**Supplementary Table 3b** 8](#_Toc46994523)

[**Supplementary Table 3c** 9](#_Toc46994524)

[**Supplementary Table 4** 9](#_Toc46994525)

[**Supplementary Table 5** 12](#_Toc46994526)

[**Supplementary Figure 1** 14](#_Toc46994527)

[**4.** **Quantitative bias analysis to assess potential confounding of HIV and COVID-19 death association by obesity** 15](#_Toc46994528)

[**Supplementary Table 7** 18](#_Toc46994529)

[**Supplementary Table 8** 20](#_Toc46994530)

[**5.** **List of contributing authors** 20](#_Toc46994531)

# **Enumeration of different health conditions from the Western Cape Provincial Health Data Centre**

The Western Cape Provincial Health Data Centre (WCPHDC) consolidates administrative, laboratory, and pharmacy data from routine electronic clinical information systems used in all public sector health facilities with linkage through a unique patient identifier. Multiple data sources are triangulated to enumerate health conditions such as diabetes mellitus (“diabetes”), hypertension, tuberculosis and HIV, with either high or moderate certainty evidence assigned for each inferred condition as outlined in Supplementary Table 1. For the main analysis we used conditions with high or moderate certainty evidence and restricted to conditions with high certainty evidence in a sensitivity analysis.

## **Supplementary Table 1**

Summary of evidences used to infer different comorbidities in the Western Cape Provincial Health Data Centre with high or moderate certainty.

| **Inferred conditions** | **High certainty** | | | |  | **Moderate** | | |
| --- | --- | --- | --- | --- | --- | --- | --- | --- |
|  | **Laboratory tests** | **Drugs dispensed** | **Procedure or ICD-10 codes** | **Other evidences** |  | **Laboratory tests** | **Drugs dispensed** | **Procedure or ICD-10 codes** |
| **HIV** | HIV-RNA viral load or positive HIV-RNA PCR or HIV genotypic resistance assay or positive HIV ELISA | valid combination antiretroviral therapy for >2 months |  | registered in HIV electronic chronic disease management system |  | CD4 count or positive rapid HIV test | 2 antiretrovirals dispensed on same day in adult | HIV  ICD-10 code (B20) |
| **Tuberculosis** | positive GeneXpert or  line probe assay or  microscopy, culture & microbiology | tuberculosis treatment regimen | tuberculosis  ICD-10 code (A15) | admitted in tuberculosis hospital;  registered in tuberculosis electronic chronic disease management system |  |  |  |  |
| **Diabetes Mellitus** | HbA1c > 6.5% or  oral glucose tolerance test >11.1mmol/l | diabetes treatment | diabetes ICD-10 code (E10/E11) |  |  | fasting blood glucose >7.0mmol/l, random blood glucose >11.0mmol/l |  |  |
| **Hypertension** |  | hydrochlorothiazide |  |  |  |  |  |  |
| **Chronic obstructive pulmonary disease /asthma** |  | β_2_ agonists e.g. salbutamol; rimiterol |  |  |  |  |  |  |
| **Chronic Kidney Disease** | 2 consecutive eGFR measures <60ml/minute/1.73m^2^,  >90 days apart | transplant medication | procedure code for kidney transplant |  |  | tissue typing prior to dialysis or transplant |  |  |
| ICD-10 International Statistical Classification of Diseases and Related Health Problems 10th Revision; eGFR estimated glomerular filtration rate | | | | | | | | |

# **Calculation of the Standardized Mortality Ratios (SMR) and population attributable fractions**

The calculation of the standardized mortality ratio follows standard methods.^1^ We define the following symbols:

$q_{g,x}$ is the COVID-19 mortality rate in HIV-negative individuals of sex *g*, aged *x*.

$a_{g,x}$ is the COVID-19 mortality rate in HIV-positive individuals of sex *g*, aged *x*.

$N_{g,x}$ is the number of HIV-negative individuals of sex *g*, aged *x*.

$H_{g,x}$is the number of HIV-positive individuals of sex *g*, aged *x*.

We further define *D_H_* to be the total number of COVID-19 deaths in HIV-positive individuals:

$$D_{H}=\sum_{g} \sum_{x} H_{g,x}a_{g,x}$$

Similarly, we define *D_T_* to be the total number of COVID-19 deaths (in both HIV-positive and HIV-negative individuals):

$$D_{T}=\sum_{g} \sum_{x} {N_{g,x}q_{g,x}+H}_{g,x}a_{g,x}$$

Estimates of the numbers of HIV-negative and HIV-positive individuals in 2020, by age and sex ($N_{g,x}$ and $H_{g,x}$), are taken from the Thembisa model for the Western Cape (version 4.3).^2^ Thembisa is a combined demographic and HIV model, developed for South Africa, and is the main model on which UNAIDS estimates for South Africa are based. The model has been calibrated to each province in South Africa, based on fitting to age-specific HIV prevalence data from household surveys and antenatal clinic surveys.^3^ The age-specific COVID-19 mortality rates are estimated by dividing the recorded numbers of deaths at each age and sex by the corresponding Thembisa estimate of the population size.

The standardized mortality ratio (SMR) is defined as the ratio of the actual number of COVID-19 deaths in the HIV-positive population to the number of COVID-19 deaths that would be expected in the HIV-positive population if their COVID-19 mortality rates were the same as in the negative population (i.e. replacing $a_{g,x}$ with $q_{g,x}$):

$$SMR=\frac{D_{H}}{\sum_{g} \sum_{x} H_{g,x}q_{g,x}}$$

The population attributable fraction (PAF) is defined as the fraction of COVID-19 deaths that are attributable HIV. This is not the same as the fraction of deaths that occur in HIV-positive individuals because some of the COVID-19 deaths in HIV-positive individuals would have occurred even if they were HIV-negative (i.e. we are interested only in the excess mortality risk in HIV-positive individuals compared to HIV-negative individuals). Mathematically,

$$PAF=\frac{\sum_{g} \sum_{x} H_{g,x}\left( a_{g,x} - q_{g,x} \right)}{D_{T}}=\frac{D_{H} - \sum_{g} \sum_{x} H_{g,x}q_{g,x}}{D_{T}}=\frac{D_{H}}{D_{T}}\left( 1 - \frac{1}{SMR} \right)$$

# **Supplementary Results**

## **Supplementary Table 2**

Characteristics of Western Cape “active patients” in public sector ≥20 years of age (public sector health care visit in last 3 years before March 1, 2020) according to COVID-19 outcome using only high certainty evidence to determine comorbidities.

|  |  | **No diagnosed COVID-19** n=3,436,810 | **COVID-19 not deceased** n=21,679 | **COVID-19 deceased** n=625 |  |
| --- | --- | --- | --- | --- | --- |
| **Sex** |  |  |  |  |  |
| **female** |  | 1,983,495 (58%) | 14,912 (69%) | 340 (54%) |  |
| **male** |  | 1,454,315 (42%) | 6,767 (31%) | 285 (46%) |  |
|  |  |  |  |  |  |
| **Age** |  |  |  |  |  |
| **20-39 years** |  | 1,913,786 (56%) | 11,640 (54%) | 46 (7%) |  |
| **40-49 years** |  | 604,976 (18%) | 4,515 (21%) | 63 (10%) |  |
| **50-59 years** |  | 447,739 (13%) | 3,227 (15%) | 162 (26%) |  |
| **60-69 years** |  | 276,082 (8%) | 1,423 (7%) | 178 (28%) |  |
| **≥70 years** |  | 194,227 (6%) | 874 (4%) | 176 (28%) |  |
|  |  |  |  |  |  |
| **Diabetes** |  |  |  |  |  |
| **none** |  | 3,177,659 (92%) | 18,616 (86%) | 256 (41%) |  |
| **diabetes HbA1c <7%** |  | 44,776 (1%) | 482 (2%) | 58 (9%) |  |
| **diabetes HbA1c 7 - 8.9%** |  | 47,199 (1%) | 582 (3%) | 94 (15%) |  |
| **diabetes HbA1c ≥9%** |  | 65,629 (2%) | 1,086 (5%) | 158 (25%) |  |
| **diabetes no HbA1c measurement** |  | 101,547 (3%) | 913 (4%) | 59 (9%) |  |
|  |  |  |  |  |  |
| **Other non-communicable diseases** |  |  |  |  |  |
| **hypertension** |  | 563,658 (16%) | 4,907 (23%) | 362 (58%) |  |
| **chronic kidney disease** |  | 61,557 (2%) | 493 (2%) | 111 (18%) |  |
| **chronic pulmonary disease / asthma** |  | 192,494 (6%) | 1,576 (7%) | 84 (13%) |  |
|  |  |  |  |  |  |
| **Tuberculosis** |  |  |  |  |  |
| **never tuberculosis** |  | 3,104,459 (90%) | 19,664 (91%) | 512 (82%) |  |
| **previous tuberculosis** |  | 281,198 (8%) | 1,698 (8%) | 87 (14%) |  |
| **current tuberculosis** |  | 51,453 (2%) | 317 (1%) | 26 (4%) |  |
|  |  |  |  |  |  |
| **HIV** |  |  |  |  |  |
| **negative** |  | 2,963,391 (86%) | 18,136 (84%) | 519 (83%) |  |
| **positive** |  | 473,419 (16%) | 3,543 (16%) | 106 (17%) |  |
|  |  |  |  |  |  |
| **VL <1000 copies/ml (last 15 mo) & ART script (last 6 mo)** |  | 240,029 (51%) | 2,314 (66%) | 71 (67%) |  |
| **VL <1000 copies/ml (2yr to 15 mo prior)  OR ART script (last 6 mo) & VL <1000 copies/ml > 2yr prior** |  | 68,865 (15%) | 409 (12%) | 11 (10%) |  |
| **VL ≥ 1000 copies/ml (last 15 mo) or CD4 <200 cells/µl (last 18 mo)** |  | 37,767 (8%) | 203 (6%) | 11 (10%) |  |
| **No VL (last 15 mo); CD4 ≥200 cells/µl or unknown (last 18 mo)** |  | 126,758 (27%) | 599 (17%) | 13 (12%) |  |
| Note: Column percentages may add up to >100% due to rounding;  HbA1c glycosylated haemoglobin; VL viral load; mo month; yr year; ART antiretroviral therapy | | | | | |

## **Supplementary Table 3a**

Current tuberculosis disease in Western Cape “active patients” in public sector ≥20 years of age (public sector health care visit in last 3 years before March 1, 2020) according to HIV status and COVID-19 outcome.

|  |  | **Public sector patients with HIV** | | |  | **Public sector patients without HIV** | | |
| --- | --- | --- | --- | --- | --- | --- | --- | --- |
|  |  | **No diagnosed COVID-19 n=536,574** | **COVID-19 not deceased n=3,863** | **COVID-19 deceased n=115** |  | **No diagnosed COVID-19 n=2,902,050** | **COVID-19 not deceased n=17,820** | **COVID-19 deceased n=510** |
| **No tuberculosis** |  |  |  |  |  |  |  |  |
| **n** |  | 512,217 | 3,691 | 99 |  | 2,872,155 | 17,675 | 500 |
| **%** |  | 95.5 | 95.6 | 86.1 |  | 99.0 | 99.2 | 98.0 |
| **Tuberculosis intensive phase  (rifampicin sensitive)** |  |  |  |  |  |  |  |  |
| **n** |  | 8,576 | 104 | 11 |  | 10,269 | 83 | 7 |
| **%** |  | 2.0 | 3.0 | 10 |  | 0.4 | 0.5 | 1.4 |
| **Tuberculosis intensive phase (rifampicin resistant)** |  |  |  |  |  |  |  |  |
| **n** |  | 678 | 7 | 3 |  | 525 | 4 | 3 |
| **%** |  | 0.1 | 0.2 | 2.6 |  | 0.0 | 0.0 | 0.6 |
| **Tuberculosis continuation (rifampicin sensitive/resistant)** |  |  |  |  |  |  |  |  |
| **n** |  | 15,103 | 61 | 2 |  | 19,101 | 58 | 0 |
| **%** |  | 2.8 | 1.6 | 1.7 |  | 0.7 | 0.3 | 0.0 |
| ^*^Tuberculosis considered intensive phase if diagnosed <3 months previously (rifampicin sensitive) or <6 months previously (rifampicin resistant). Patients were considered to have “current tuberculosis” if first evidence of tuberculosis episode was between March 1, 2019 and study closure (June 9, 2020) and, in COVID-19 cases, <30 days after COVID-19 diagnosis date. All tuberculosis diagnoses after March 1, 2020 in COVID-19 cases were <30 days after date of COVID-19 diagnosis. | | | | | | | | |

## **Supplementary Table 3b**

Multivariate hazard ratios (HRs) and 95% confidence intervals (CI) for associations with COVID-19 death from Cox-proportional hazards models among all “active patients” in the public sector ≥20 years of age (public sector health visit in last 3 years before March 1, 2020) (n=3,460,932). Current tuberculosis is categorized as rifampicin sensitive or resistant and separated into intensive and continuation phases of treatment.

|  | **Adjusted HR** | **95% CI** | **p-value** |  |
| --- | --- | --- | --- | --- |
| **Sex** |  |  |  |  |
| **female** | Ref |  |  |  |
| **male** | 1.45 | 1.24; 1.70 | <0.001 |  |
| **Age** |  |  |  |  |
| **20-39 years** | Ref |  |  |  |
| **40-49 years** | 2.84 | 1.93; 4.18 | <0.001 |  |
| **50-59 years** | 7.85 | 5.56; 11.08 | <0.001 |  |
| **60-69 years** | 11.64 | 8.18; 16.57 | <0.001 |  |
| **≥70 years** | 16.96 | 11.81; 24.35 | <0.001 |  |
| **Diabetes** |  |  |  |  |
| **none** | Ref |  |  |  |
| **diabetes HbA1c <7%** | 5.34 | 3.94; 7.23 | <0.001 |  |
| **diabetes HbA1c 7 - 8.9%** | 8.51 | 6.59; 11.0 | <0.001 |  |
| **diabetes HbA1c ≥9%** | 11.99 | 9.63; 14.92 | <0.001 |  |
| **diabetes no HbA1c measurement** | 2.91 | 2.18; 3.89 | <0.001 |  |
| **Other non-communicable diseases** |  |  |  |  |
| **hypertension** | 1.31 | 1.09; 1.57 | 0.004 |  |
| **chronic kidney disease** | 0.92 | 0.73; 1.16 | 0.494 |  |
| **chronic pulmonary disease / asthma** | 1.86 | 1.49; 2.32 | <0.001 |  |
| **Tuberculosis** |  |  |  |  |
| **never tuberculosis** | Ref |  |  |  |
| **tuberculosis intensive phase (rifampicin sensitive)^*^** | 5.54 | 3.44; 8.93 | <0.001 |  |
| **tuberculosis intensive phase (rifampicin resistant)^*^** | 26.54 | 11.79; 59.75 | <0.001 |  |
| **tuberculosis continuation phase (rifampicin sensitive/resistant)** | 0.33 | 0.08; 1.31 | 0.115 |  |
| **previous tuberculosis** | 1.49 | 1.17; 1.91 | 0.001 |  |
| **HIV** |  |  |  |  |
| **negative** | Ref |  |  |  |
| **positive** | 2.14 | 1.70; 2.70 | <0.001 |  |
| ^*^Tuberculosis considered intensive phase if diagnosed <3 months previously (rifampicin sensitive) or <6 months previously (rifampicin resistant). Patients were considered to have “current tuberculosis” if first evidence of tuberculosis episode was between March 1, 2019 and study closure (June 9, 2020) and, in COVID-19 cases, <30 days after COVID-19 diagnosis date. All tuberculosis diagnoses after March 1, 2020 in COVID-19 cases were <30 days after date of COVID-19 diagnosis. HbA1c glycosylated haemoglobin; HR hazard ratio; CI confidence interval | | | | |
|  |  |  |  |  |
|  |  |  |  |  |

## **Supplementary Table 3c**

Multivariate hazard ratios (HRs) and 95% confidence intervals (CI) for associations with COVID-19 death from Cox-proportional hazards models among all “active patients” in the public sector ≥20 years of age (public sector health visit in last 3 years before March 1, 2020) (n=3,460,932). Current tuberculosis is categorized as microbiologically confirmed or not.

| **Description of data used in analysis** | **Current tuberculosis categorized as microbiologically confirmed or not** | | |
| --- | --- | --- | --- |
|  | **Adjusted HR** | **95% CI** | **p-value** |
| **Sex** |  |  |  |
| **female** | Ref |  |  |
| **male** | 1.45 | 1.23; 1.70 | <0.001 |
| **Age** |  |  |  |
| **20-39 years** | Ref |  |  |
| **40-49 years** | 2.83 | 1.92; 4.16 | <0.001 |
| **50-59 years** | 7.79 | 5.52; 10.99 | <0.001 |
| **60-69 years** | 11.56 | 8.12; 16.45 | <0.001 |
| **≥70 years** | 16.82 | 11.71; 24.16 | <0.001 |
| **Diabetes** |  |  |  |
| **none** | Ref |  |  |
| **diabetes HbA1c <7%** | 5.37 | 3.96; 7.27 | <0.001 |
| **diabetes HbA1c 7 - 8.9%** | 8.52 | 6.60; 11.01 | <0.001 |
| **diabetes HbA1c ≥9%** | 12.05 | 9.69; 14.99 | <0.001 |
| **diabetes no HbA1c measurement** | 2.91 | 2.18; 3.89 | <0.001 |
| **Other non-communicable diseases** |  |  |  |
| **hypertension** | 1.31 | 1.09; 1.57 | 0.004 |
| **chronic kidney disease** | 1.86 | 1.49; 2.33 | <0.001 |
| **chronic pulmonary disease / asthma** | 0.93 | 0.73; 1.17 | 0.516 |
| **Tuberculosis** |  |  |  |
| **never tuberculosis** | Ref |  |  |
| **previous tuberculosis** | 1.52 | 1.19; 1.94 | 0.001 |
| **current tuberculosis (microbiologically confirmed)** | 3.16 | 1.94; 5.15 | <0.001 |
| **current tuberculosis (not microbiologically confirmed)** | 2.12 | 1.09; 4.13 | 0.026 |
| **HIV** |  |  |  |
| **negative** | Ref |  |  |
| **positive** | 2.14 | 1.70; 2.70 | <0.001 |
| HR hazard ratio; CI confidence interval; HbA1c glycosylated haemoglobin. | | | |

## **Supplementary Table 4**

Multivariate hazard ratios (HRs) and 95% confidence intervals (CI) for associations with COVID-19 death from Cox-proportional hazards models among all “active patients” in public sector (age ≥20 years with a public sector health visit in the last 3 years before March 1, 2020) (n=3,460,932). Sensitivity analyses were conducted as follows: (i) as per main analysis but not adjusted for location; (ii) as per main analysis but including follow up through June 16, 2020; (iii) with competing risks regression; (iv) considering only comorbidities inferred with high certainty evidence (n=3,459,114); (v) classifying patients as viraemic or immunosuppressed based on viral load and CD4 count results in the last month only; (vi) restricted to patients with ≥1 visit per year for last 3 years (n=991,969).

| **Description of data used in analysis** | **Not adjusted for location, censored on 9 June 2020 to allow for 7 day delay in reporting of deaths** | | | | **Adjusted for location,  all follow-up through  16 June 2020** | | | | **Adjusted for location, censored on 9 June 2020 to allow for 7 day delay in reporting of deaths** | | | | **Adjusted for location,  censored on 9 June 2020 to allow for 7 day delay in reporting of deaths** | | | | **Adjusted for location,  censored on 9 June 2020 to allow for 7 day delay in reporting of deaths** | | | | **Adjusted for location, restricted to 991,969 patients with ≥1 visit per year for last 3 years; censored on 9 June 2020 to allow for 7 day delay in reporting of deaths** | | | |
| --- | --- | --- | --- | --- | --- | --- | --- | --- | --- | --- | --- | --- | --- | --- | --- | --- | --- | --- | --- | --- | --- | --- | --- | --- |
| **Certainty of evidence for comorbidities** | **All certainty** | | | | **All certainty** | | | | **All certainty** | | | | **High certainty** | | | | **All certainty; Restricted definition of viraemia / immunosuppression in PLWH** | | | | **All certainty** | | | |
| **Analysis method** | **Cox-proportional hazards** | | | | **Cox-proportional hazards** | | | | **Competing risks, non-COVID-19 death as competing risk** | | | | **Cox-proportional hazards** | | | | **Cox-proportional hazards** | | | | **Cox-proportional hazards** | | | |
|  | **aHR** | **95% CI** | **p-value** | **aHR** | | **95% CI** | **p-value** | **aHR** | | **95% CI** | **p-value** | **aHR** | | **95% CI** | **p-value** | **aHR** | | **95% CI** | **p-value** | **aHR** | | **95% CI** | **p-value** |  |
| **Sex** |  |  |  |  | |  |  |  | |  |  |  | |  |  |  | |  |  |  | |  |  |  |
| **female** | Ref |  |  | Ref | |  |  | Ref | |  |  | Ref | |  |  | Ref | |  |  | Ref | |  |  |  |
| **male** | 1.41 | 1.20-1.65 | <0.001 | 1.53 | | 1.33-1.75 | <0.001 | 1.45 | | 1.23-1.70 | <0.001 | 1.46 | | 1.24-1.71 | <0.001 | 1.45 | | 1.24-1.70 | <0.001 | 1.51 | | 1.23-1.86 | <0.001 |  |
| **Age** |  |  |  |  | |  |  |  | |  |  |  | |  |  |  | |  |  |  | |  |  |  |
| **20-39 years** | Ref |  |  | Ref | |  |  | Ref | |  |  | Ref | |  |  | Ref | |  |  | Ref | |  |  |  |
| **40-49 years** | 2.67 | 1.82-3.92 | <0.001 | 3.21 | | 2.32-4.45 | <0.001 | 2.83 | | 1.92-4.17 | <0.001 | 2.83 | | 1.93-4.16 | <0.001 | 2.77 | | 1.89-4.08 | <0.001 | 2.21 | | 1.20-4.05 | 0.011 |  |
| **50-59 years** | 7.12 | 5.05-10.05 | <0.001 | 7.59 | | 5.63-10.21 | <0.001 | 7.78 | | 5.47-11.06 | <0.001 | 7.83 | | 5.55-11.05 | <0.001 | 7.72 | | 5.47-10.89 | <0.001 | 6.35 | | 3.68-10.94 | <0.001 |  |
| **60-69 years** | 10.69 | 7.51-15.2 | <0.001 | 10.89 | | 8.03-14.78 | <0.001 | 11.51 | | 7.97-16.62 | <0.001 | 11.62 | | 8.17-16.54 | <0.001 | 11.60 | | 8.15-16.51 | <0.001 | 10.03 | | 5.77-17.44 | <0.001 |  |
| **≥70 years** | 15.35 | 10.69-22.02 | <0.001 | 15.66 | | 11.45-21.42 | <0.001 | 16.72 | | 11.38-24.56 | <0.001 | 16.97 | | 11.82-24.37 | <0.001 | 16.88 | | 11.75-24.24 | <0.001 | 13.08 | | 7.41-23.11 | <0.001 |  |
| **Diabetes** |  |  |  |  | |  |  |  | |  |  |  | |  |  |  | |  |  |  | |  |  |  |
| **none** | Ref |  |  | Ref | |  |  | Ref | |  |  | Ref | |  |  | Ref | |  |  | Ref | |  |  |  |
| **diabetes HbA1c <7%** | 5.25 | 3.88-7.11 | <0.001 | 4.71 | | 3.59-6.19 | <0.001 | 5.35 | | 3.88-7.37 | <0.001 | 5.29 | | 3.91-7.17 | <0.001 | 5.31 | | 3.92-7.19 | <0.001 | 3.44 | | 2.36-5.02 | <0.001 |  |
| **diabetes HbA1c 7 - 8.9%** | 8.57 | 6.64-11.07 | <0.001 | 8.00 | | 6.39-10.02 | <0.001 | 8.50 | | 6.44-11.22 | <0.001 | 8.36 | | 6.47-10.79 | <0.001 | 8.47 | | 6.56-10.93 | <0.001 | 5.59 | | 4.07-7.66 | <0.001 |  |
| **diabetes HbA1c ≥9%** | 12.53 | 10.08-15.58 | <0.001 | 11.70 | | 9.69-14.14 | <0.001 | 12.03 | | 9.47-15.28 | <0.001 | 11.92 | | 9.59-14.81 | <0.001 | 12.07 | | 9.70-15.01 | <0.001 | 8.42 | | 6.42-11.05 | <0.001 |  |
| **diabetes   no HbA1c measurement** | 3.07 | 2.3-4.1 | <0.001 | 2.88 | | 2.24-3.70 | <0.001 | 2.92 | | 2.15-3.96 | <0.001 | 2.78 | | 2.07-3.73 | <0.001 | 2.95 | | 2.20-3.94 | <0.001 | 2.80 | | 1.90-4.14 | <0.001 |  |
| **Other non-communicable diseases** |  |  |  |  | |  |  |  | |  |  |  | |  |  |  | |  |  |  | |  |  |  |
| **hypertension** | 1.49 | 1.24-1.78 | <0.001 | 1.30 | | 1.11-1.52 | <0.001 | 1.31 | | 1.08-1.59 | 0.007 | 1.31 | | 1.09-1.57 | 0.004 | 1.30 | | 1.09-1.56 | 0.004 | 1.44 | | 1.12-1.85 | 0.004 |  |
| **chronic kidney disease** | 1.93 | 1.54-2.41 | <0.001 | 1.89 | | 1.56-2.30 | <0.001 | 1.85 | | 1.45-2.36 | <0.001 | 1.87 | | 1.50-2.34 | <0.001 | 1.86 | | 1.49-2.33 | <0.001 | 2.15 | | 1.67-2.78 | <0.001 |  |
| **chronic pulmonary  disease or asthma** | 0.94 | 0.75-1.19 | 0.631 | 1.08 | | 0.89-1.32 | 0.423 | 0.92 | | 0.73-1.17 | 0.498 | 0.94 | | 0.74-1.18 | 0.573 | 0.93 | | 0.73-1.17 | 0.525 | 0.92 | | 0.70-1.21 | 0.558 |  |

| **Tuberculosis** |  |  |  |  |  |  |  |  |  |  |  |  |  |  |  |  |  |  |  |
| --- | --- | --- | --- | --- | --- | --- | --- | --- | --- | --- | --- | --- | --- | --- | --- | --- | --- | --- | --- |
| **never tuberculosis** | Ref |  |  | Ref |  |  | Ref |  |  | Ref |  |  | Ref |  |  | Ref |  |  |  |
| **previous tuberculosis** | 1.53 | 1.2-1.95 | <0.001 | 1.46 | 1.18-1.81 | <0.001 | 1.51 | 1.18-1.94 | 0.001 | 1.45 | 1.13-1.86 | 0.004 | 2.55 | 1.69-3.83 | <0.001 | 1.78 | 1.32-2.41 | 0.004 |  |
| **current tuberculosis** | 2.68 | 1.79-4 | 0.001 | 2.67 | 1.88-3.80 | <0.001 | 2.64 | 1.78-3.91 | <0.001 | 2.42 | 1.57-3.74 | <0.001 | 1.49 | 1.16-1.91 | 0.002 | 1.72 | 0.91-3.26 | 0.096 |  |
| **HIV** |  |  |  |  |  |  |  |  |  |  |  |  |  |  |  |  |  |  |  |
| **negative** | Ref |  |  | Ref |  |  | Ref |  |  | Ref |  |  |  |  |  | Ref |  |  |  |
| **positive** | 2.62 | 2.09-3.29 | <0.001 | 1.94 | 1.58-2.39 | <0.001 | 2.13 | 1.69-2.70 | <0.001 | 2.26 | 1.78-2.88 | <0.001 |  |  |  | 1.56 | 1.14-2.15 | 0.006 |  |
|  |  |  |  |  |  |  |  |  |  |  |  |  |  |  |  |  |  |  |  |
| **VL <1000 copies/ml  (last 15 mo)   & ART script   (last 6 mo)^a^** |  |  |  |  |  |  |  |  |  | 2.62 | 1.99-3.45 | <0.001 | 2.61 | 1.98-3.44 | 0.000 | 1.79 | 1.27-2.54 | 0.001 |  |
| **VL <1000 copies/ml   (2yr to 15 mo prior) OR   ART script (last 6 mo))  & VL <1000 copies/ml  (>2yr prior)** |  |  |  |  |  |  |  |  |  | 1.77 | 0.97-3.26 | 0.064 | 1.77 | 0.96-3.24 | 0.067 | 0.39 | 0.10-1.59 | 0.191 |  |
| **VL ≥1000 copies/ml  (last 15 mo)  OR CD4 <200 cells/µl  (last 18 mo)** |  |  |  |  |  |  |  |  |  | 3.33 | 1.78-6.24 | <0.001 |  |  |  | 2.85 | 1.29-6.29 | 0.010 |  |
| **VL ≥1000 copies/ml  (last 12 mo)  OR CD4 <200 cells/µl   (last 12 mo)** |  |  |  |  |  |  |  |  |  |  |  |  | 3.59 | 1.96-6.56 | 0.000 |  |  |  |  |
| **No VL (last 15 mo);   CD4 ≥200 cells/µl   or unknown  (last 18 mo)** |  |  |  |  |  |  |  |  |  | 1.31 | 0.75-2.30 | 0.345 |  |  |  | 1.15 | 0.51-2.59 | 0.743 |  |
| **No VL (last 12 mo);   CD4 ≥200 cells/µl  or unknown   (last 12 mo)** |  |  |  |  |  |  |  |  |  |  |  |  | 1.31 | 0.84-2.05 | 0.241 |  |  |  |  |
| ^a^Reference category for hazard ratio is HIV negative; adjusted for all other variables listed in this table in a model that included the listed categories of HIV viral load (VL), antiretroviral therapy (ART) and immunosuppression instead of the binary variable HIV positive vs negative; the effect of the other variables on mortality was similar to those presented here. aHR adjusted hazard ratio; SHR sub-distribution hazard ratio; CI confidence interval; HbA1c glycosylated haemoglobin; VL viral load; mo month(s); yr year(s); ART antiretroviral therapy | | | | | | | | | | | | | | | | | | | |

## **Supplementary Table 5**

Multivariate hazard ratios (HRs) and 95% confidence intervals (CI) for associations with COVID-19 death from Cox-proportional hazards models among all adult COVID-19 cases diagnosed through to study closure (June 9, 2020) including period after June 1, 2020 when testing criteria changed (n=19,868).

| **Description of data used in analysis** |  | **Including all COVID-19 cases diagnosed until study closure (9 June 2020)** | | |
| --- | --- | --- | --- | --- |
| **Certainty of evidence for comorbidities** |  | **All certainty** | | |
|  |  | **Adjusted HR** | **95% CI** | **p-value** |
| **Sex** |  |  |  |  |
| **female** |  | Ref |  |  |
| **male** |  | 1.47 | 1.25; 1.72 | <0.001 |
| **Age** |  |  |  |  |
| **20-39 years** |  |  |  |  |
| **40-49 years** |  | 2.70 | 1.83; 3.98 | <0.001 |
| **50-59 years** |  | 8.72 | 6.17; 12.31 | <0.001 |
| **60-69 years** |  | 19.05 | 13.37; 27.13 | <0.001 |
| **≥70 years** |  | 31.43 | 21.93; 45.05 | <0.001 |
| **Diabetes** |  |  |  |  |
| **none** |  |  |  |  |
| **diabetes HbA1c <7%** |  | 2.54 | 1.89; 3.43 | <0.001 |
| **diabetes HbA1c 7 - 8.9%** |  | 3.48 | 2.70; 4.48 | <0.001 |
| **diabetes HbA1c ≥9%** |  | 3.96 | 3.19; 4.91 | <0.001 |
| **diabetes no HbA1c measurement** |  | 2.07 | 1.55; 2.76 | <0.001 |
| **Other non-communicable diseases** |  |  |  |  |
| **hypertension** |  | 1.07 | 0.89; 1.27 | 0.488 |
| **chronic kidney disease** |  | 1.81 | 1.45; 2.25 | <0.001 |
| **chronic pulmonary disease / asthma** |  | 0.83 | 0.66; 1.05 | 0.125 |
| **Tuberculosis** |  |  |  |  |
| **never tuberculosis** |  |  |  |  |
| **previous tuberculosis** |  | 1.53 | 1.20; 1.95 | 0.001 |
| **current tuberculosis** |  | 1.78 | 1.19; 2.66 | 0.005 |
| **HIV** |  |  |  |  |
| **negative** |  |  |  |  |
| **positive** |  | 1.75 | 1.40; 2.19 | <0.001 |
|  |  |  |  |  |
| **VL <1000 copies/ml (last 15 mo) & ART script (last 6 mo)^a^** |  | 1.75 | 1.34; 2.29 | <0.001 |
| **VL <1000 copies/ml (2yr to 15 mo prior)  OR ART script (last 6 mo) & VL <1000 copies/ml >2yr prior** |  | 1.59 | 0.87; 2.92 | 0.135 |
| **VL ≥ 1000 copies/ml (last 15 mo) or CD4 <200 cells/µl (last 18 mo)** | | 3.80 | 2.07; 6.95 | <0.001 |
| **No VL (last 15 mo); CD4 ≥200 cells/µl or unknown (last 18 mo)** |  | 1.54 | 1.01; 2.33 | 0.042 |
|  |  |  |  |  |
| **ART in PLWH with script issued in last 12 months^b^** |  |  |  |  |
| **abacavir or zidovudine** |  | Ref |  |  |
| **tenofovir disoproxil fumarate** |  | 0.49 | 0.27; 0.86 | 0.014 |
| **efavirenz** |  | Ref |  |  |
| **lopinavir** |  | 0.80 | 0.34; 1.86 | 0.601 |
| **atazanavir** |  | 0.86 | 0.26; 2.87 | 0.806 |
| **dolutegravir** |  | 0.71 | 0.24; 2.09 | 0.530 |
| **ART duration** |  |  |  |  |
| **<1 year** |  | Ref |  |  |
| **1-2 years** |  | 0.78 | 0.25; 2.47 | 0.676 |
| **≥2 years** |  | 0.47 | 0.20; 1.12 | 0.089 |
| ^a^Reference category is HIV negative; adjusted for all other variables listed in this table in a model that included the listed categories of HIV viral load (VL), antiretroviral therapy (ART) and immunosuppression instead of the binary variable HIV positive vs negative; the effect of the other variables on mortality was similar to those presented here; ^c^Restricted to patients with documented antiretrovirals dispensed in the last 12 months, adjusted for all other variables listed in this table in a model that included the relevant antiretrovirals and ART duration; the effect of the other variables on mortality was similar similar to those presented here. HR hazard ratio; CI confidence interval; HbA1c glycosylated haemoglobin; ART antiretroviral therapy; mo months; yr years;  PLWH people living with HIV | | | | |

**Supplementary Table 6**

Multivariate hazard ratios (HRs) and 95% confidence intervals (CI) for associations with COVID-19 death from Cox-proportional hazards models among all adult COVID-19 cases diagnosed before June 1, 2020 with current tuberculosis categorized as rifampicin sensitive or resistant and separated into intensive and continuation phase (n=15,203).

| **Description of data used in analysis** |  | **Limited to cases diagnosed before 1 June 2020 when testing criteria changed;  rifampicin sensitive vs resistant tuberculosis and intensive/continuation phases separated** | | |
| --- | --- | --- | --- | --- |
| **Certainty of evidence for comorbidities** |  | **All certainty** | | |
|  |  | **Adjusted HR** | **95% CI** | **p-value** |
| **Sex** |  |  |  |  |
| **female** |  | Ref |  |  |
| **male** |  | 1.48 | 1.26; 1.74 | <0.001 |
| **Age** |  |  |  |  |
| **20-39 years** |  |  |  |  |
| **40-49 years** |  | 2.68 | 1.82; 3.96 | <0.001 |
| **50-59 years** |  | 8.68 | 6.14; 12.25 | <0.001 |
| **60-69 years** |  | 18.84 | 13.22; 26.85 | <0.001 |
| **≥70 years** |  | 31.14 | 21.73; 44.63 | <0.001 |
| **Diabetes** |  |  |  |  |
| **none** |  |  |  |  |
| **diabetes HbA1c <7%** |  | 2.54 | 1.88; 3.42 | <0.001 |
| **diabetes HbA1c 7 - 8.9%** |  | 3.51 | 2.72; 4.53 | <0.001 |
| **diabetes HbA1c ≥9%** |  | 3.93 | 3.17; 4.87 | <0.001 |
| **diabetes no HbA1c measurement** |  | 2.08 | 1.56; 2.78 | <0.001 |
| **Other non-communicable diseases** |  |  |  |  |
| **hypertension** |  | 1.06 | 0.89; 1.27 | 0.518 |
| **chronic kidney disease** |  | 1.81 | 1.45; 2.25 | <0.001 |
| **chronic pulmonary disease / asthma** |  | 0.85 | 0.67; 1.07 | 0.172 |
| **Tuberculosis** |  |  |  |  |
| **never tuberculosis** |  |  |  |  |
| **previous tuberculosis** |  | 1.48 | 1.16; 1.89 | 0.001 |
| **tuberculosis intensive phase (rifampicin sensitive)*** |  | 1.99 | 1.20; 3.30 | 0.008 |
| **tuberculosis intensive phase (rifampicin resistant)*** |  | 5.55 | 2.25; 13.68 | <0.001 |
| **tuberculosis continuation phase (rifampicin sensitive/resistant)** | | 0.88 | 0.36; 2.14 | 0.783 |
| **HIV** |  |  |  |  |
| **negative** |  |  |  |  |
| **positive** |  | 1.78 | 1.42; 2.22 | <0.001 |
| *Tuberculosis considered intensive phase if diagnosed <3 months previously (rifampicin sensitive) or <6 months previously (rifampicin resistant). Patients were considered to have “current tuberculosis” if first evidence of tuberculosis episode was between March 1, 2019 and study closure (June 1, 2020) (COVID-19 non-cases) and <30 days after COVID-19 diagnosis date (COVID-19 cases). All tuberculosis diagnoses after March 1, 2020 in COVID-19 cases were <30 days after date of COVID-19 diagnosis. HR hazard ratio; CI confidence interval; HbA1c glycosylated haemoglobin. | | | | |

## **Supplementary Figure 1**

Prior CD4 and viral load history among 70 people living with HIV (PLWH) hospitalized with COVID-19 in whom CD4 count was <200 cells/µl during COVID-19 episode. Grey blocks indicate patients with prior history indicating that they had CD4 ≥200 cells/µl or were stable on ART.
y years; VL viral load; mo months; ART antiretroviral therapy


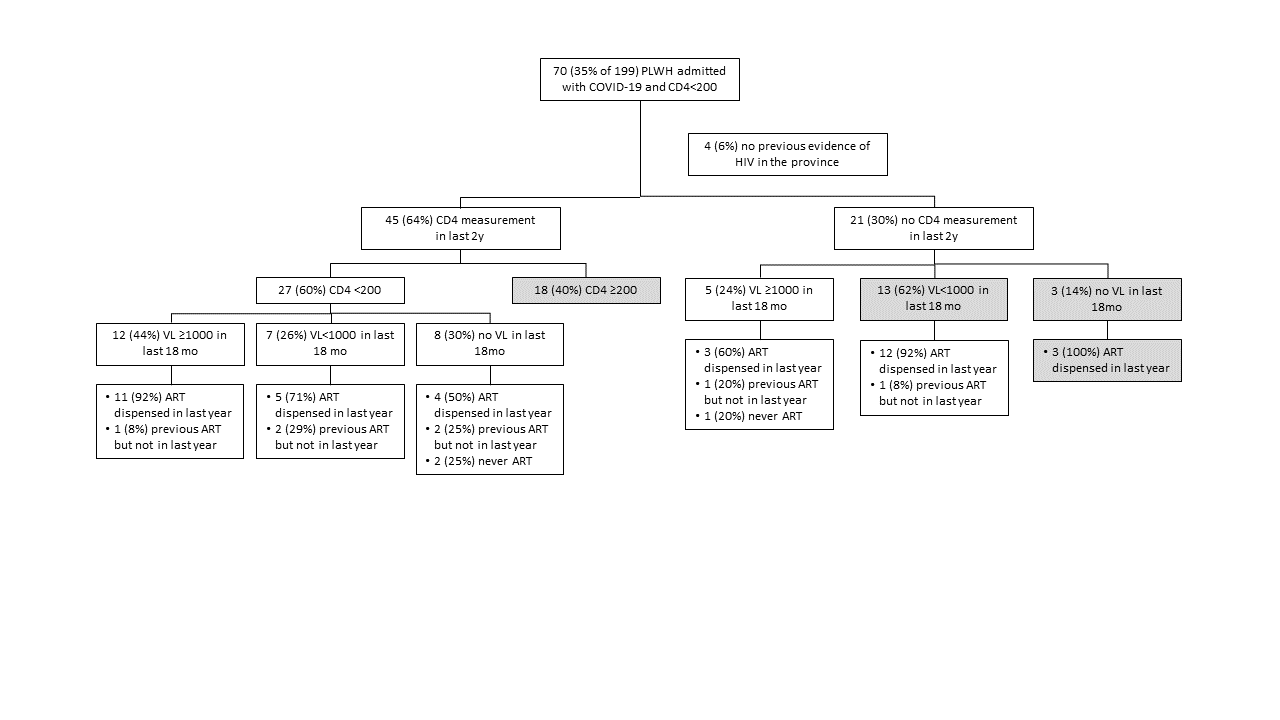


# **Quantitative bias analysis to assess potential confounding of HIV and COVID-19 death association by obesity**

The objective of the quantitative bias analysis is to assess the extent to which the measured association between HIV and COVID-19 mortality might be biased due to not controlling for obesity (a variable that is not routinely measured in the Western Cape Provincial Health Data Centre). Supplementary Figure 1 illustrates the conceptual model. We distinguish between untreated and treated HIV for the purpose of this analysis, as the prevalence of obesity is likely to be different in treated and untreated HIV-positive individuals, which in turn has implications for the extent of the bias if we do not control for obesity.

Supplementary Figure 1: Potential causal pathways linking HIV and COVID-19 mortality

We adopt a Bayesian approach to quantitative bias analysis, which involves specifying prior distributions to represent the plausible ranges of uncertainty around the key parameters in the above model.^4^ Using notation similar to that in Greenland,^5^ we define the parameters in which we are interested as follows:

- Ω(*U*) is the odds of obesity in the HIV-negative population.
- Ω(*T*_1_*U*) is the odds ratio for the association between untreated HIV and obesity.
- Ω(*T*_2_*U*) is the odds ratio for the association between treated HIV and obesity.
- Ω(*UY*|*T*) is the odds ratio for the association between obesity and COVID-19 death, when controlling for HIV.
- Ω(*T*_1_*Y*|*U*) is the odds ratio for the association between untreated HIV and COVID-19 mortality, when controlling for obesity.
- Ω(*T*_2_*Y*|*U*) is the odds ratio for the association between treated HIV and COVID-19 mortality, when controlling for obesity.
- Ω(*T*_1_*Y*) is the odds ratio for the association between untreated HIV and COVID-19 mortality, when not controlling for obesity.
- Ω(*T*_2_*Y*) is the odds ratio for the association between treated HIV and COVID-19 mortality, when not controlling for obesity.

We define *B*_1_ as the bias due to not controlling for obesity when assessing the association between untreated HIV and COVID-19 mortality:

$$B_{1}=\frac{\text{Ω}\left( T_{1}Y \right)}{\text{Ω}\left( T_{1}Y | U \right)}$$

When Ω(*T*_1_*Y*|*U*) is unknown (as is the case here), the bias can instead be calculated using the following formula^5^:

$$B_{1}=\frac{\left( \text{Ω}\left( T_{1}U \right)\text{Ω}\left( UY|T \right)\text{Ω}\left( U \right)+1 \right)\left( \text{Ω}\left( U \right)+1 \right)}{\left( \text{Ω}\left( T_{1}U \right)\text{Ω}\left( U \right)+1 \right)\left( \text{Ω}\left( UY|T \right)\text{Ω}\left( U \right)+1 \right)}$$

Substituting this estimate of *B*_1_ into the previous equation allows us to estimate Ω(*T*_1_*Y*|*U*). Similarly, *B*_2_ is defined as the bias due to not controlling for obesity when assessing the association between treated HIV and COVID-19 mortality, and is estimated in the same way.

The sections that follow describe the approaches to specifying prior distributions for each of the key parameters.

**Odds of obesity in the HIV negative population (Ω(*U*))**

We analysed data from the 2016 Demographic and Health Survey (DHS).^6^ In this survey, the prevalence of obesity (a BMI of 30 or higher) in HIV-negative adults (aged 15 or older) in the Western Cape was 35.3% (95% CI: 29.0-41.6%). If *P* is the prevalence of obesity, then the odds of obesity is Ω(*U*) = *P*/(1 – *P*). We assign a normal distribution to represent the uncertainty in *P*, with a mean of 35.3% and a standard deviation of 3.2% (to be consistent with the 95% confidence intervals around the survey estimate).

**Association between HIV and obesity (Ω(*T*_1_*U*) and Ω(*T*_2_*U*))**

We assessed the association between HIV and obesity using data from the 2016 DHS.^6^ We fitted a logistic regression model to the data collected for adults (ages 15 and older), controlling for age and sex. For the purpose of this analysis, individuals were classified as having treated HIV if they were HIV-positive and reported taking one or more antiretroviral drugs (this is likely to be an underestimate of the true ART uptake as many ART patients do not report being on treatment or else report not knowing their HIV status).^7-10^

Results are summarized in Supplementary Table 7. These results suggest that obesity is reduced in untreated HIV-positive individuals, and is further reduced in treated HIV-positive individuals (although the difference in odds of obesity between treated and untreated HIV is not statistically significant). These results may appear counter-intuitive, as previous cohort studies suggest that ART initiation leads to significant weight recovery.^11^ However, the results are consistent with the results from another large South African survey in KwaZulu-Natal, which found that in multivariable analysis, BMI was lower in untreated HIV-positive individuals than in HIV-negative individuals and that BMI was even lower in patients who were on ART.^12^ Cohort studies typically compare BMI immediately prior to ART initiation (usually when patients already have advanced HIV) and BMI at longer ART durations, which may give a distorted sense of the average levels of weight loss in untreated HIV.

## **Supplementary Table 7**

Factors associated with obesity in the 2016 Demographic and Health Survey

|  | Adjusted odds ratio (95% CI) |
| --- | --- |
| HIV status |  |
| HIV-negative | 1 |
| HIV-positive, untreated | 0.62 (0.53-0.73) |
| HIV-positive, treated | 0.47 (0.37-0.61) |
| Sex |  |
| Male | 1 |
| Female | 5.56 (4.81-6.42) |
| Age group |  |
| 15-24 | 1 |
| 25-34 | 3.85 (3.12-4.74) |
| 35-44 | 7.72 (6.20-9.61) |
| 45-54 | 8.41 (6.75-10.48) |
| 55-64 | 8.21 (6.54-10.29) |
| 65+ | 5.36 (4.27-6.72) |

To represent the uncertainty around Ω(*T*_1_*U*) and Ω(*T*_2_*U*), we use lognormal prior distributions with means and standard deviations matching the odds ratios in Supplementary Table 7.

**Association between obesity and COVID-19 mortality (Ω(*UY*|*T*))**

A large study based on NHS data in the UK found that among COVID-19 cases, obesity was strongly associated with mortality, with the increase being modest for grade I obesity (aHR 1.27, 95% CI: 1.18-1.36), higher for grade II obesity (aHR 1.56, 95% CI: 1.41-1.73) and highest for grade III obesity (aHR 2.27, 95% CI: 1.99-2.58).^13^ In the South African 2016 DHS, the proportion of obese adults in each class was 55%, 26% and 19% respectively. Applying these proportions to the odds ratios in the UK study, the weighted average odds ratio is 1.53. Although the UK study did not directly control for HIV, it did control for “immunosuppressive conditions” (unspecified), and controlling for HIV would probably not have changed the association between obesity and COVID-19 mortality given the low HIV prevalence in the UK.

We assign a lognormal prior distribution to represent the uncertainty around Ω(*UY*|*T*). The prior distribution has a mean of 1.53 (the same as the weighted average of the odds ratios in the UK study) and 2.5 and 97.5 percentiles of 1.39 and 1.69 respectively. These percentiles correspond the weighted averages of the lower and upper confidence interval limits for the odds ratios quoted previously.

**Monte Carlo analysis**

We approximate Ω(*T*_1_*Y*|*U*) and Ω(*T*_2_*Y*|*U*) by randomly drawing 10,000 parameter combinations from the prior distributions specified previously, and substituting into the previous equations to solve for Ω(*T*_1_*Y*|*U*) and Ω(*T*_2_*Y*|*U*). We also randomly draw values from the estimates for Ω(*T*_1_*Y*) and Ω(*T*_2_*Y*) in Table 4 of the main paper (1.73 (95% CI: 1.10-2.71) and 1.60 (95% CI:1.19-2.17) respectively), again using lognormal distributions. Table 2 summarizes the results of this Monte Carlo analysis. Controlling for obesity leads to a slight increase in the estimated strength of association between HIV and COVID-19 mortality, which is because the prevalence of obesity is lower in HIV-positive individuals than in HIV-negative individuals.

## **Supplementary Table 8**

Bias due to not controlling for obesity

|  | Untreated HIV | | Treated HIV | |
| --- | --- | --- | --- | --- |
|  | Symbol | Estimate | Symbol | Estimate |
| Bias due to not controlling for  obesity | *B*_1_ | 0.956  (0.937-0.973) | *B*_2_ | 0.934  (0.909-0.958) |
| OR for association between HIV  and COVID-19 mortality | Ω(*T*_1_*Y*) | 1.73  (1.10-2.73) | Ω(*T*_2_*Y*) | 1.60  (1.19-2.17) |
| OR for association between HIV  and COVID-19 mortality, adjusted | Ω(*T*_1_*Y*\|*U*) | 1.85  (1.14-2.84) | Ω(*T*_1_*Y*\|*U*) | 1.74  (1.27-2.34) |

# **List of contributing authors**

**Western Cape Government: Health**

*Health Impact Assessment Directorate:* Andrew Boulle,^1,2^ Mary-Ann Davies,^1,2^
Hannah Hussey,^1,3^ Muzzammil Ismail,^1,3^ Erna Morden,^1,3^ Ziyanda Vundle,^1,4^
Virginia Zweigenthal^1,3^*Metro and Rural Health Outbreak Response Team Leads*: Hassan Mahomed,^4,5^
Masudah Paleker,^4,5^ David Pienaar,^6^ Yamanya Tembo^3, 6^*Communicable Disease Control sub-Directorate*: Charlene Lawrence,^7^ Washiefa Isaacs,^7^ Hlengani Mathema^7,8^*Provincial Health Data Centre:* Derick Allen,^2^ Taryn Allie,^1,2^ Jamy-Lee Bam,^1^
Kasturi Buddiga,^1,2^ Pierre Dane,^1,2^ Alexa Heekes,^1,2^ Boitumelo Matlapeng,^1,2^
Themba Mutemaringa,^1,2^ Luckmore Muzarabani,^1,2^ Florence Phelanyane,^1,2^ Rory Pienaar,^1^ Catherine Rode,^1,2^ Mariette Smith,^1,2^ Nicki Tiffin,^1, 2,9,10^ Nesbert Zinyakatira^1,3^
*Health Programmes Directorate*: Carol Cragg,^11^ Frederick Marais,^11,12^ Vanessa Mudaly,^3,11^ Jacqueline Voget^11^*Hospitals: George Hospital*: Jody Davids,^13^ Francois Roodt,^13^ Nellis van Zyl Smit,^13^
Alda Vermeulen^13^*Groote Schuur Hospital*: Kevin Adams,^14,15^ Gordon Audley,^14,16^ Kathleen Bateman,^14,16^
Peter Beckwith,^14,16^ Marc Bernon,^14,15^ Dirk Blom,^14,16^ Linda Boloko,^14,16^ Jean Botha,^14,16^ Adam Boutall,^14,15^ Sean Burmeister,^14,15^ Lydia Cairncross,^14,15^ Gregory Calligaro,^14,16^
Cecilia Coccia,^14,16^ Chadwin Corin,^14,16^ Remy Daroowala,^14,15^ Joel A. Dave,^14,16^
Elsa De Bruyn,^14,16^ Martin De Villiers,^14,16^ Mimi Deetlefs,^14,16^ Sipho Dlamini,^14,16^
Thomas Du Toit,^14,16^ Wilhelm Endres,^14,16^ Tarin Europa,^14,16^ Graham Fieggan,^14,15^
Anthony Figaji,^14,15^ Petro Frankenfeld,^14,16^ Elizabeth Gatley,^14,16^ Phindile Gina,^14,16^
Evashan Govender,^14,16^ Rochelle Grobler,^14,16^ Manqoba Vusumuzi Gule,^14,16^
Christoff Hanekom,^14,16^ Michael Held,^14,16^ Alana Heynes,^14,16^ Sabelo Hlatswayo,^14,16^
Bridget Hodkinson,^14,16^ Jeanette Holtzhausen,^14^ Shakeel Hoosain,^14,16^Ashely Jacobs,^14,16^ Miriam Kahn,^14,15^ Thania Kahn,^14,16^ Arvin Khamajeet,^14,15^ Joubin Khan,^14,16^ Riaasat Khan^14,16^ Alicia Khwitshana,^14,16^ Lauren Knight,^14,16^ Sharita Kooverjee,^14,16^ Rene Krogscheepers,^14,16^ Jean Jacque Kruger,^14,16^ Suzanne Kuhn,^14,16^ Kim Laubscher,^14,15^ John Lazarus,^14,15^
Jacque Le Roux,^14,16^ Scott Lee Jones,^14,16^ Dion Levin,^14,16^ Gary Maartens,^14,16^
Thina Majola,^14,16^ Rodgers Manganyi,^14,16^ David Marais,^14,16^ Suzaan Marais,^14,16^
Francois Maritz,^14,15^ Deborah Maughan,^14,16^ Simthandile Mazondwa,^14,16^Luyanda Mbanga,^14,16^ Nomonde Mbatani,^14,16^ Bulewa Mbena,^14,16^ Graeme Meintjes,^14,16^ Marc Mendelson,^14,16^ Ernst Möller,^14,16^ Allison Moore,^14,15^ Babalwa Ndebele,^14,16^
Marc Nortje,^14,15^ Ntobeko Ntusi,^14,16^ Funeka Nyengane,^14,16^ Chima Ofoegbu,^14,15^Nectarios Papavarnavas,^14,16^ Jonny Peter,^14,16^ Henri Pickard,^14,15^ Kent Pluke,^14,15^
Peter J. Raubenheimer,^14,16^ Gordon Robertson,^14,16^ Julius Rozmiarek,^14,16^ A Sayed,^14,16^ Matthias Scriba,^14,15^ Hennie Sekhukhune,^14,16^ Prasun Singh,^14,16^ Elsabe Smith,^14,16^
Vuyolwethu Soldati,^14,15^ Cari Stek,^14,16^ Robert van den berg,^14,16^ Le Roux van der Merwe,^14,16^ Pieter Venter,^14,15^ Barbra Vermooten,^14,16^ Gerrit Viljoen,^14,16^ Santhuri Viranna,^14,16^Jonno Vogel,^14,16^ Nokubonga Vundla,^14,16^ Sean Wasserman,^14,16^ Eddy Zitha^14,16^*Helderberg Hospital*: Vanessa Lomas-Marais,^17^ Annie Lombard,^17^ Katrin Stuve,^17^
Werner Viljoen^17^*Karl Bremer Hospital*: De Vries Basson,^18^ Sue Le Roux,^18^ Ethel Linden-Mars,^18^
Lizanne Victor,^18^ Mark Wates,^18^ Elbe Zwanepoel^18^*Khayelitsha District Hospital*: Nabilah Ebrahim,^19^ Sa'ad Lahri,^19^ Ayanda Mnguni^19^
*Mitchells Plain Hospital*: Thomas Crede,^20^ Martin de Man,^20,31^ Katya Evans,^20,31^
Clint Hendrikse,^20,31^ Jonathan Naude,^20^ Moosa Parak,^20,31^ Patrick Szymanski,^20^Candice Van Koningsbruggen^20, 31^*Tygerberg Hospital*: Riezaah Abrahams,^21,22^ Brian Allwood,^21,22^ Christoffel Botha,^21,22^ Matthys Henndrik Botha,^21,23^ Alistair Broadhurst,^21,22^ Dirkie Claasen,^21,22^ Che Daniel,^21,22^ Riyaadh Dawood,^21,22^ Marie du Preez,^21,22^ Nicolene Du Toit,^21,23^  Kobie Erasmus,^21,24^Coenraad F. N. Koegelenberg,^21,22^ Shiraaz Gabriel,^21,22^ Susan Hugo,^21,22^ Thabiet Jardine,^21,22^ Clint Johannes,^21,22^ Sumanth Karamchand,^21,22^ Usha Lalla,^21,22^ Eduard Langenegger,^21,23^Eize Louw,^21,22^ Boitumelo Mashigo,^21,22^ Nonte Mhlana,^21,22^ Chizama Mnqwazi,^21,22^
Ashley Moodley,^21,23^ Desiree Moodley,^21,22^ Saadiq Moolla,^21,22^ Abdurasiet Mowlana,^21,22^ Andre Nortje,^21,22^ Elzanne Olivier,^21,23^ Arifa Parker,^21,22^ Chané Paulsen,^21,22^
Hans Prozesky,^21,22^ Jacques Rood,^21,22^ Tholakele Sabela,^21,22^ Neshaad Schrueder,^21,22^ Nokwanda Sithole,^21,22^ Sthembiso Sithole,^21,22^ Jantjie J. Taljaard,^21,22^ Gideon Titus,^21,22^
Tian Van Der Merwe,^21,23^ Marije van Schalkwyk,^21,22^ Luthando Vazi,^21,22^
Abraham J Viljoen,^21,22^ Mogamat Yazied Chothia^21,22^
*Emergency Medical Services*: Vanessa Naidoo,^24^ Lee Alan Wallis^24,31^*District Outbreak Response Teams:* Mumtaz Abbass,^25^ Juanita Arendse,^25^ Rizqa Armien,^25^
Rochelle Bailey,^25^ Muideen Bello,^25^ Rachel Carelse,^25^ Sheron Forgus,^25^ Nosi Kalawe,^25^
Saadiq Kariem,^25^ Mariska Kotze,^25^ Jonathan Lucas,^25^ Juanita McClaughlin,^25^
Kathleen Murie,^25^ Leilah Najjaar,^25^ Liesel Petersen,^25^ James Porter,^25^ Melanie Shaw,^25^ Dusica Stapar,^25^ Michelle Williams^25^

**City of Cape Town:** Linda Aldum,^26^ Natacha Berkowitz,^26^ Raakhee Girran,^26^ Kevin Lee,^26^ Lenny Naidoo,^26^ Caroline Neumuller^26^

**Outbreak Response Pod Team:** Kim Anderson,^2^ Kerrin Begg,^3^ Lisa Boerlage,^3^
Morna Cornell,^2^ Renée de Waal,^2^ Lilian Dudley,^5^ René English,^5^ Jonathan Euvrard,^2^
Pam Groenewald,^27^ Nisha Jacob,^3^ Heather Jaspan,^32^ Emma Kalk,^2^ Naomi Levitt,^16^
Thoko Malaba,^3^ Patience Nyakato,^2^ Gabriela Patten,^2^ Helen Schneider,^28^
Maylene Shung King,^3^ Priscilla Tsondai,^2^ James Van Duuren,^3^ Nienke van Schaik

**National Institute for Communicable Diseases**: Lucille Blumberg,^8,33^ Cheryl Cohen,^8,34^
Nelesh Govender,^8,29^ Waasila Jassat,^8^ Tendesayi Kufa,^8^ Kerrigan McCarthy,^8^ Lynn Morris^8,35^

**National Health Laboratory Service**: Nei-yuan Hsiao,^30^ Ruan Marais^30^

**Statistical and Analytic Support**: Jon Ambler,^9^ Olina Ngwenya,^9^ Richard Osei-Yeboah,^10^ Leigh Johnson,^2^ Reshma Kassanjee,^2^ Tsaone Tamuhla^10^

**Affiliations**

| ^1^ | Health Impact Assessment, Western Cape Government: Health |
| --- | --- |
| ^2^ | Centre for Infectious Disease Epidemiology and Research, School of Public Health and Family Medicine, University of Cape Town |
| ^3^ | School of Public Health and Family Medicine, University of Cape Town |
| ^4^ | Division of Health Systems and Public Health, Department of Global Health, Faculty of Medicine and Health Sciences, Stellenbosch University |
| ^5^ | Metro Health Services, Western Cape Government: Health |
| ^6^ | Rural Health Services, Western Cape Government: Health |
| ^7^ | Communicable Disease Sub-Directorate, Western Cape Government: Health |
| ^8^ | National Institute for Communicable Diseases, National Health Laboratory Service, South Africa |
| ^9^ | Wellcome Centre for Infectious Disease Research in Africa, University of Cape Town |
| ^10^ | Division of Computational Biology, University of Cape Town |
| ^11^ | Health Programmes Directorate, Western Cape Government: Health |
| ^12^ | Faculty of Health Sciences, North West University |
| ^13^ | George Hospital, Western Cape Government: Health |
| ^14^ | Groote Schuur Hospital, Western Cape Government: Health |
| ^15^ | Department of Medicine, University of Cape Town |
| ^16^ | Department of Surgery, University of Cape Town |
| ^17^ | Department of Radiology, University of Cape Town |
| ^18^ | Karl Bremer Hospital, Western Cape Government: Health |
| ^19^ | Khayelitsha District Hospital, Western Cape Government: Health |
| ^20^ | Mitchells Plain and Heideveld Hospitals, Western Cape Government: Health |
| ^21^ | Tygerberg Hospital, Western Cape Government: Health |
| ^22^ | Department of Medicine, Stellenbosch University |
| ^23^ | Department of Obstetrics and Gyneacology, Stellenbosch University |
| ^24^ | Emergency Medical Services, Western Cape Government: |
| ^25^ | Western Cape Government: Health |
| ^26^ | City Health, Community Services and Health, City of Cape Town |
| ^27^ | South African Medical Research Council Burden of Disease Research Unit |
| ^28^ | School of Public Health, University of the Western Cape |
| ^29^ | School of Pathology, University of the Witwatersrand and School of Pathology, University of Cape Town |
| ^30^ | National Health Laboratory Service and Division of Virology, School of Pathology, University of Cape Town |
| ^31^ | Division of Emergency Medicine, University of Cape Town |
| ^32^ | Division of Immunology and Institute of Infectious Diseases and Molecular Medicine, University of Cape Town |
| ^33^ | University of Pretoria |
| ^34^ | School of Public Health, University of Witwatersrand |
| ^35^ | University of Witwatersrand, South African Medical Research Council Antibody Immunity Research Unit and the Centre for the AIDS Programme in South Africa (CAPRISA) |

**References**

1. Armitage P. Statistical Methods in Medical Research. Oxford, United Kingdom: Blackwell; 1971.

2. Johnson LF, Dorrington RE. Modelling the impact of HIV in South Africa’s provinces: 2020 update: Centre for Infectious Disease Epidemiology and Research, University of Cape Town; 2020. Available at: https://www.thembisa.org/. Accessed 26 June 2020.

3. Johnson LF, Dorrington RE, Moolla H. HIV epidemic drivers in South Africa: a model-based evaluation of factors accounting for inter-provincial differences in HIV prevalence and incidence trends. South African Journal of HIV Medicine 2017;18:a695.

4. Lash TL, Fox MP, MacLehose RF, Maldonado G, McCandless LC, Greenland S. Good practices for quantitative bias analysis. International Journal of Epidemiology 2014;43:1969-85.

5. Greenland S. Multiple-bias modelling for analysis of observational data. J Roy Stat Soc A 2005;168:267-306.

6. Department of Health, Statistics South Africa, South African Medical Research Council, ICF. South Africa Demographic and Health Survey 2016. Pretoria; 2019. Available at: https://www.dhsprogram.com/pubs/pdf/FR337/FR337.pdf. Accessed 19 March 2019.

7. Grobler A, Cawood C, Khanyile D, Puren A, Kharsany ABM. Progress of UNAIDS 90-90-90 targets in a district in KwaZulu-Natal, South Africa, with high HIV burden, in the HIPSS study: a household-based complex multilevel community survey. Lancet HIV 2017;4:e505-e13.

8. Johnson LF, Rehle TM, Jooste S, Bekker LG. Rates of HIV testing and diagnosis in South Africa, 2002-2012: successes and challenges. AIDS 2015;1401-9.

9. Rohr JK, Xavier Gomez-Olive F, Rosenberg M, et al. Performance of self-reported HIV status in determining true HIV status among older adults in rural South Africa: a validation study. J Int AIDS Soc 2017;20:21691.

10. Sandfort TGM, Dominguez K, Kayange N, et al. HIV testing and the HIV care continuum among sub-Saharan African men who have sex with men and transgender women screened for participation in HPTN 075. PloS ONE 2019;14:e0217501.

11. Nduka CU, Uthman OA, Kimani PK, Stranges S. Body fat changes in people living with HIV on antiretroviral therapy. AIDS Reviews 2016;18:198-211.

12. Malaza A, Mossong J, Bärnighausen T, Newell ML. Hypertension and obesity in adults living in a high HIV prevalence rural area in South Africa. PloS ONE 2012;7:e47761.

13. Williamson E, Walker AJ, Bhaskaran K, et al. OpenSAFELY: factors associated with COVID-19-related hospital death in the linked electronic health records of 17 million adult NHS patients. Nature 2020. doi: 10.1038/s41586-020-2521-4.
